# Two-way communication between SecY and SecA suggests a Brownian ratchet mechanism for protein translocation

**DOI:** 10.7554/eLife.15598

**Published:** 2016-05-16

**Authors:** William John Allen, Robin Adam Corey, Peter Oatley, Richard Barry Sessions, Steve A Baldwin, Sheena E Radford, Roman Tuma, Ian Collinson

**Affiliations:** 1 School of Biochemistry, University of Bristol, Bristol, United Kingdom; 2 Astbury Centre for Structural Molecular Biology, University of Leeds, Leeds, United Kingdom; 3 School of Biomedical Sciences, University of Leeds, Leeds, United Kingdom; 4 School of Molecular and Cellular Biology, University of Leeds, Leeds, United Kingdom; MRC Laboratory of Molecular Biology, United Kingdom

**Keywords:** protein secretion, protein translocation, single molecule FRET, molecular dynamics, SecYEG, SecA, *E. coli*

## Abstract

The essential process of protein secretion is achieved by the ubiquitous Sec machinery. In prokaryotes, the drive for translocation comes from ATP hydrolysis by the cytosolic motor-protein SecA, in concert with the proton motive force (PMF). However, the mechanism through which ATP hydrolysis by SecA is coupled to directional movement through SecYEG is unclear. Here, we combine all-atom molecular dynamics (MD) simulations with single molecule FRET and biochemical assays. We show that ATP binding by SecA causes opening of the SecY-channel at long range, while substrates at the SecY-channel entrance feed back to regulate nucleotide exchange by SecA. This two-way communication suggests a new, unifying 'Brownian ratchet' mechanism, whereby ATP binding and hydrolysis bias the direction of polypeptide diffusion. The model represents a solution to the problem of transporting inherently variable substrates such as polypeptides, and may underlie mechanisms of other motors that translocate proteins and nucleic acids.

**DOI:** http://dx.doi.org/10.7554/eLife.15598.001

## eLife digest

A protective membrane surrounds all cells, and controls what goes in and out of the cell. Many proteins that are made inside the cell need to be exported in order to do their job. In most organisms, a specialised transport motor that sits inside the membrane, known as 'Sec', carries out this export process. Sec recognises proteins that need to be exported and pushes them across the membrane and out of the cell. The energy required to do this comes from the cell's universal power source, a molecule called ATP.

Previous studies have shown what Sec looks like, but not how it pushes proteins from one side of the membrane to the other. Currently, the most popular theory for how Sec works is that it grabs hold of part of the protein and pushes it through a gate in the membrane. It then lets go and goes back to grab and push the next bit of the protein.

Allen, Corey, Oatley et al. have now used a combination of experimental and computational methods to look at how the different parts of Sec move around as it uses ATP. The reasoning behind using these methods was that it's easier to understand how a motor works by watching it in action rather than just looking at a still picture.

Using these methods, Allen, Corey, Oatley et al. show that the biggest movement in Sec as it uses ATP is in the membrane gate itself, which opens and closes. This suggests that Sec acts like a turnstile: proteins can freely move one way across the membrane, but are prevented from moving back in again. This mechanism has not been described before and may apply to other transport systems.

Further investigations will be needed to understand exactly how Sec recognises cargo and starts the transport process, and to explore the specific features of a protein that activate the turnstile. It also remains to be discovered how this transport process differs in other, non-bacterial cells. This could potentially help us develop new drugs that specifically block the bacterial Sec system without affecting human cells.

**DOI:** http://dx.doi.org/10.7554/eLife.15598.002

## Introduction

The Sec system is the main pathway for protein secretion in all forms of life. At the translocon core is a hetero-trimeric membrane protein complex – SecYEG in the plasma membrane of prokaryotes and Sec61αγβ in the ER of eukaryotes – which forms a protein channel across the membrane (Ito et al., 1983; Brundage et al., 1990; Görlich et al., 1992). Pre-protein substrates with an N-terminal signal sequence are targeted to the translocon in an unfolded state (Arkowitz et al., 1993), whereupon they are threaded across (secretion) or transferred laterally into the membrane (insertion). Protein translocation can occur either co-translationally following emergence of the nascent chain from the ribosome, or post-translationally. In prokaryotes, secretion is mostly post-translational (Hartl et al., 1990): substrates are recognised by the cytosolic ATPase SecA (Lill et al., 1989), which targets them to SecYEG and then drives them through the protein channel using energy derived from ATP binding and hydrolysis, and the trans-membrane proton motive force (PMF) (Brundage et al., 1990).

**Figure 1.**
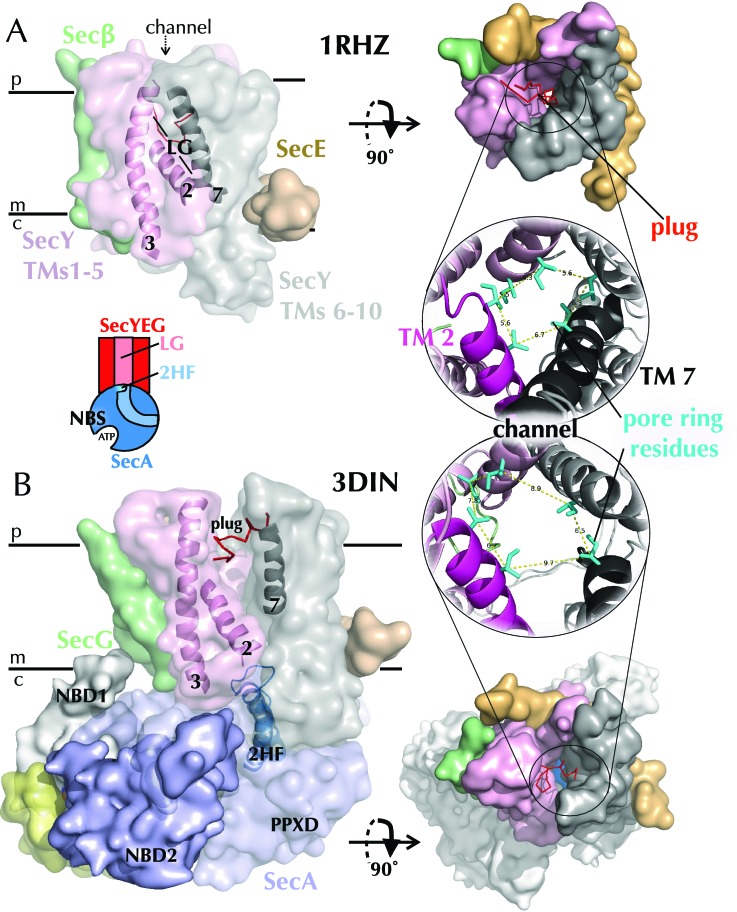
Structures of SecYEG. (**A**) Surface representations of SecYEβ from *M. janaschii*; PDB code 1RHZ (Van den Berg et al., 2004) viewed from the side (left) and periplasm (top right). TMs 1–5 of SecY are shown in pink and TMs 6–10 in grey, with the LG helices (TMs 2, 3 and 7) highlighted as cartoons in the side view. SecE is shown in orange, SecG/β in green and the plug (helix 2a) as red ribbons. Also shown (bottom right) is a cartoon closeup of the central channel of SecYEβ (from the periplasm) with the plug removed and the six residues comprising the pore ring shown as turquoise sticks. TMs 2 and 7 of SecY are highlighted in magenta and black, respectively. (**B**) As in panel A but the structure of SecYEG-SecA from *Thermotoga maritima*, PDB code 3DIN (Zimmer et al., 2008). SecA is shown in pale blue, with the 2HF highlighted bright blue, NBD1 in white, NBD2 blue and a region of SecA unique to thermophilic organisms in yellow. A schematic of the SecYEG-SecA complex is also shown (centre-left of figure), with SecYEG in red, SecA in blue and the key domains marked. This scheme is used as a reference throughout the paper. **DOI:** http://dx.doi.org/10.7554/eLife.15598.003

A structure of SecYEβ from *Methanococcus jannaschii* provided the first glimpse of the trans-membrane channel through which secretory proteins pass (Van den Berg et al., 2004) (PDB code 1RHZ; Figure 1A). SecY consists of ten trans-membrane helices (TMs), which form a ‘clamshell’ structure in the membrane, with five TMs on either side of the central protein channel, blocked by a ‘plug’ and a seal comprising six hydrophobic side chains (Figure 1A). This conformation is reinforced on one side by SecE, while on the opposite side a lateral gate (LG) connects the central protein-conducting channel to the lipid bilayer (Figure 1A). Thus, opening of the ‘clamshell’ could facilitate passage of secretory proteins through the central channel across the membrane, as well as the partitioning of membrane proteins *via* the LG into the bilayer.

SecA is a cytoplasmic ATPase that drives protein translocation through SecYEG (Lill et al., 1989; Brundage et al., 1990; Akimaru et al., 1991). The ATP turnover cycle of SecA is directly coupled to protein translocation (Economou and Wickner, 1994; Karamanou et al., 1999), but as yet the mechanism by which this is accomplished is unclear. Structural studies have revealed that SecA consists of two RecA-like nucleotide binding domains (NBD1 and NBD2), which together form the nucleotide-binding site (NBS) (Hunt et al., 2002) (Figure 1B). A ‘two-helix finger’ (2HF), at the SecY binding interface (Zimmer et al., 2008), and a pre-protein cross-linking domain (PPXD) both make contacts with the translocating polypeptide (Bauer and Rapoport, 2009).

A breakthrough in our understanding of how SecYEG and SecA operate came with a crystal structure of a complex of the two from *T. maritima* (Zimmer et al., 2008). SecY and SecA interact tightly, with the 2HF from SecA protruding into the SecY channel, and a long loop between TMs 6–7 of SecY buried inside SecA (Figure 1B). The structure also revealed conformational changes within both SecYEG and SecA. In SecA, a large movement of the PPXD activates the ATPase (Gold et al., 2013) and is thought to clamp the substrate in place, while in SecYEG the LG opens, causing a widening of the channel through which translocating substrates pass (Figure 1B) (Zimmer et al., 2008). Presumably, these changes prime the complex for intercalation of the pre-protein: however, they represent only a single snapshot in the dynamic process of protein translocation, regulated ultimately by the binding and hydrolysis of ATP. More recently, several electron microscopy structures have been solved for the Sec machinery in complex with the ribosome and nascent chains (Park et al., 2014; Gogala et al., 2014; Pfeffer et al., 2015; Voorhees and Hegde, 2016). However, whilst these structures reveal key features of the interactions between components, they do not provide mechanistic insights of how protein translocation *per se* occurs. Here, we take a different approach: using all-atom molecular dynamics (MD) simulations, we identify key structural differences between the ATP- and ADP-bound complexes, and combine these with biochemical and single molecule FRET analyses. Together, the results allow us to propose a model for ATP-dependent transport, in which two-way communication between the NBS of SecA and the channel of SecY biases the direction of random substrate diffusion, in a 'Brownian ratchet' mechanism.

## Results

### Molecular dynamics reveals nucleotide-dependent structural changes in SecY

**Figure 2.**
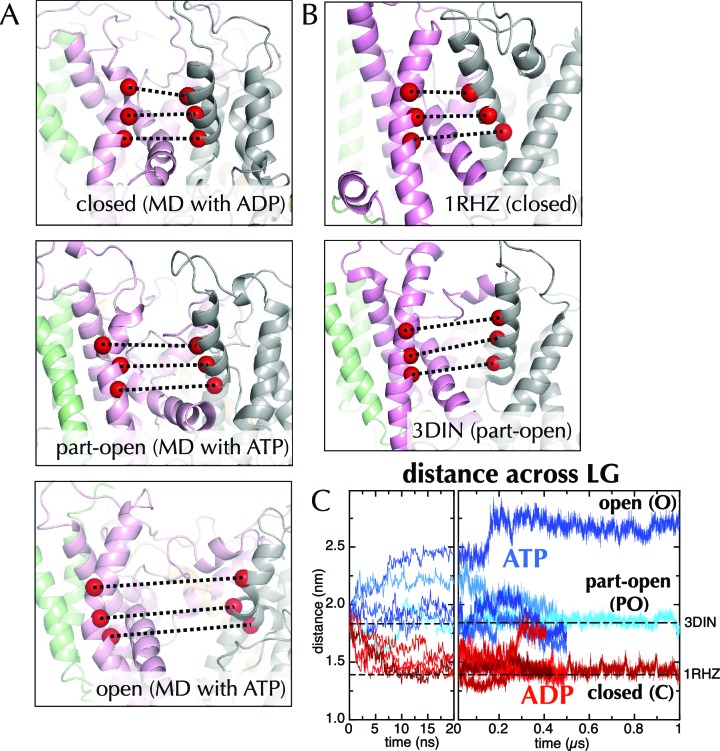
Molecular dynamics reveals nucleotide-dependent changes in the lateral gate of SecY. (**A**) 1 µs snapshots of the closed (top; after MD with ADP), part-open (middle, ATP) and open (bottom, ATP) forms of SecYEG, viewed side-on into the LG. TMs 1–5 of SecY are shown in pink, TMs 6–10 in grey, and SecG in green. Three residue pairs across the LG are shown (red spheres; residues 124/275, 127/278 and 130/282 in *T. maritima* numbering), with the distances between them drawn out as dotted black lines. Note that in the OPLS-AA force field, ATP and AMPPNP are essentially indistinguishable. (**B**) As in panel A, but showing the closed LG of SecYEβ (*M. janaschii*; PDB code 1RHZ (van den Berg et al., 2004); top) and the part-open LG of SecYEG-SecA (*T. maritima*, PDB code 3DIN (Zimmer et al., 2008); bottom). (**C**) Average distances between the residue pairs in panel B across all 10 MD simulations, with the initial 20 ns expanded for clarity. The input (3DIN) and the resting translocon (1RHZ) distances are also indicated. ATP simulations are shown in blue and ADP simulations in red, with representative open (O), part open (PO) and closed (C) trajectories indicated. **DOI:** http://dx.doi.org/10.7554/eLife.15598.004

The structure of SecYEG-SecA was previously determined bound to a non-hydrolysable, non-natural nucleotide, ADP-BeF_x_ (Figure 1B; PDB code 3DIN) (Zimmer et al., 2008). ADP-BeF_x_ has been reported to mimic ATP in a pre-hydrolysis state (Fisher et al., 1995; Phan et al., 1997), however it has also been shown to produce post-hydrolysis intermediate states (Peyser et al., 2001). In addition, the presence of the fluoroberyllate ion has been known to produce artefacts (Henry et al., 1993). We reasoned that changing the bound nucleotide to either an ATP or ADP molecule *in silico* would help illuminate the two key states of the SecA hydrolytic cycle. To achieve this, complexes were built using the protein and Mg^2+^ atoms from 3DIN, but with either ADP or ATP in place of ADP-BeF_x _(Piggot et al., 2012). The resultant structures were embedded in a POPC membrane (Ulmschneider and Ulmschneider, 2009) and five MD simulations of between 0.4–1 µs were run for each state (Figure 2 and Figure 2—figure supplement 1).

**Figure 2—figure supplement 1.**
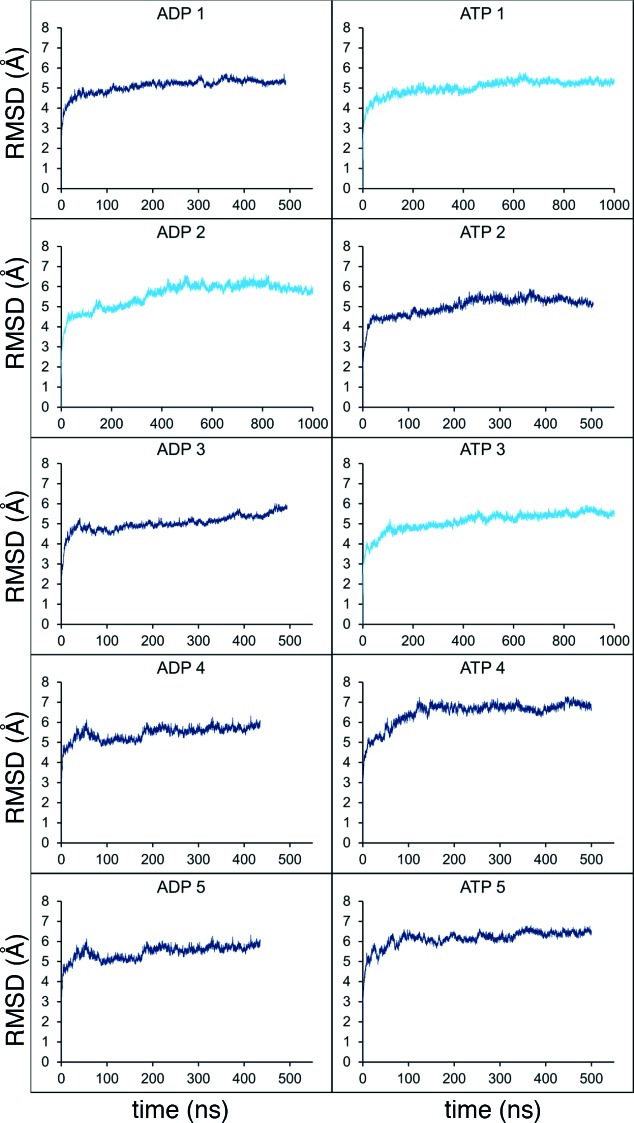
Molecular dynamics stability. Stability analyses of the MD simulations, measuring the root-mean-squared-deviation (RMSD) of the protein Cα atoms across the trajectory. Shown are the analyses for all 10 simulations from Figure 2C (5 x ADP and 5 x ATP), with the shorter simulations in dark blue, and those extended to 1 µs in light blue. **DOI:** http://dx.doi.org/10.7554/eLife.15598.005

**Figure 2—figure supplement 2.**
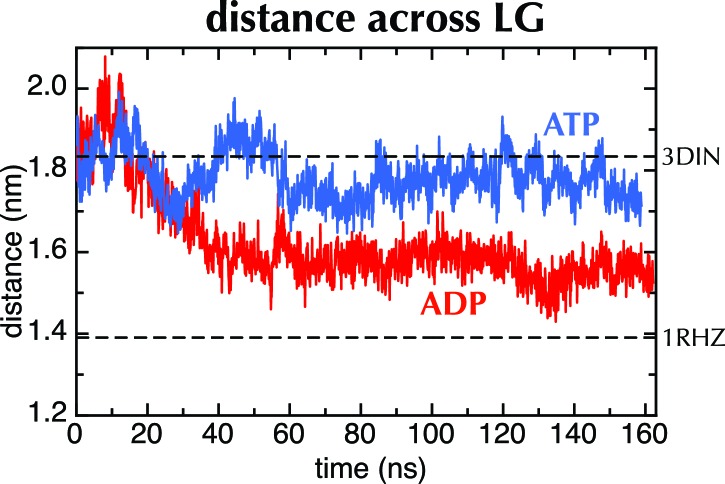
MD with Amber force field. Distance across the LG as a function of time for ATP (blue) and ADP (red) as in Figure 2C, but carried out with a different force field (Amber ff99SB-ILDN). As with OPLS-AA, ADP leads to closure of the LG, while with ATP it remains in a part open conformation. **DOI:** http://dx.doi.org/10.7554/eLife.15598.006

When ADP-BeF_x_ was replaced with ATP, the LG either retained its part-open ('PO') state of about 1.8 nm (Figure 2A, middle panel, compare to Figure 2B, lower panel; Figure 2C) or widened further to ~2.6 nm forming an open state ('O'; Figure 2A, lower panel; Figure 2C). Conversely, substitution of ADP-BeF_x _with ADP resulted in LG closure in all five simulations to ~1.5 nm ('C'; Figure 2A, upper panel; Figure 2C). Remarkably, this closed state closely resembles a previous structure of the resting SecY-complex in the absence of SecA (1RHZ (Van den Berg et al., 2004); Figure 2B, upper panel and Figure 2C). The distance over which this conformational change is propagated – over 5 nm from the NBS to the LG – is considerable. Note that simulations using a different molecular mechanics force field (Amber ff99SB-ILDN; Figure 2—figure supplement 2) show a similar effect.

**Figure 3.**
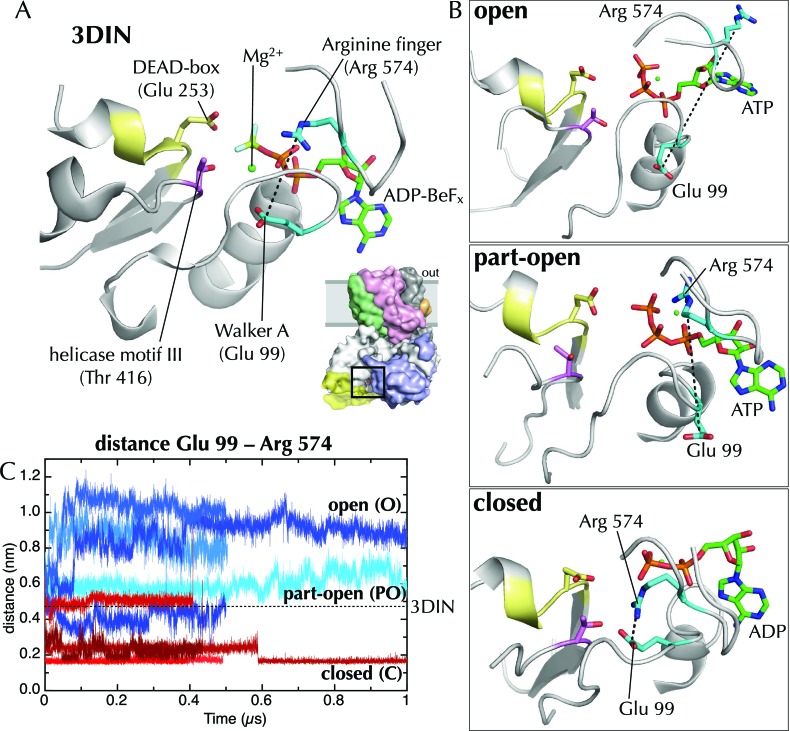
Effects of ATP and ADP on the nucleotide binding site. (**A**) Closeup of the NBS of SecA in the *T. maritima* SecYEG-SecA structure (3DIN; [Zimmer et al., 2008]).The ADP-BeF_x_ moiety is shown as sticks (carbon green, nitrogen blue, oxygen red, phosphorus orange, beryllium lime and fluorine pale blue), and key helicase loops are shown as white cartoons. Conserved helicase features are shown as coloured sticks and numbered as in *T. maritima*: Glu99 of Walker A (helicase motif Ia); Glu253 of Walker B (DEAD box of helicase motif II); Thr416 of helicase motif III; and Arg574 (‘arginine finger’) of helicase motif VI. A black dotted line marks the distance between Arg574 and Glu99. (**B**) The same view as in panel A, but for post-microsecond simulation snapshots in the open, part-open and closed simulations. Of note are Arg574 and Glu99, which remain distant in the open and part-open simulations (ATP), but come close enough to form a salt bridge in the closed simulation (ADP). In helicases, this conserved arginine finger on motif VI has been shown to couple ATPase activity with substrate binding (Hall et al., 1998), underlining its importance in SecA. After the Arg574 to Glu99 salt bridge has formed in the ADP simulations, the Walker A motif is bought into contact with a conserved threonine (Thr416) in helicase motif III, which in the helicase family has been shown to use ATP binding to create an RNA binding site (Banroques et al., 2010). This threonine communicates directly with the SecA DEAD box—providing a likely route forsignal transmission of ATP hydrolysis away from the NBS towards the polypeptide binding sites. (**C**) Minimum distance analysis between residues Glu99 and Arg574 of SecA across the simulation data confirms that a Glu99-Arg574 interaction is formed in 4/5 of the ADP simulations, whereas it is absent in all 5 ATP simulations. Interestingly, the ADP simulation that does not find this salt bridge is the same simulation in which the SecY LG samples the part-open state (Figure 2C). Thus, it appears that nucleotide dependent formation and breaking of the Glu99-Arg574 salt bridge is involved in the coupling process leading ultimately to the respective closure and opening of the channel. **DOI:** http://dx.doi.org/10.7554/eLife.15598.007

The canonical helicase motifs within the NBS of SecA – particularly the conserved arginine finger in helicase motif VI, which coordinates the β-phosphate of the bound nucleotide (Pause et al., 1993) – respond differently to ATP and ADP (Figure 3). In this system the configuration of these residues correlates to the open or closed state of the channel (Figure 3C). Along with additional conformational changes (detailed in the legend to Figure 3), these data suggest a clear route of signal transduction from the SecA NBS to the polypeptide binding sites. However, the rest of the pathway within SecA is too subtle to be apparent from the MD data. Nonetheless, the initial opening or closing event is rapid: all the ADP and ATP trajectories diverge within the first 10 ns (Figure 2C). The speed of this suggests that the input model represents a high-energy intermediate – probably due to the presence of ADP-BeF_x_.

### A FRET system monitors lateral gate opening

**Figure 4.**
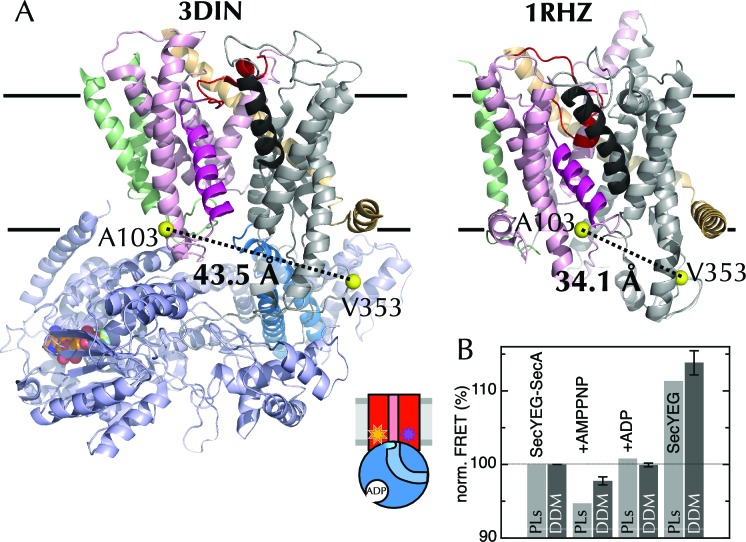
The extrinsic FRET pair of SecY**EG reports on the distance across the lateral gate. (**A**) Structures of SecYEG-SecA from *T. maritima* (left; part-open; PDB code 3DIN [Zimmer et al., 2008]) and SecYEβ from *M. jannaschii* (right; closed; PDB code 1RHZ [Van den Berg et al., 2004]). TMs 1–5 of SecY are coloured pink (TM2 highlighted magenta), TMs 6–10 grey (TM7 highlighted black), SecE orange, SecG/β green and SecA pale blue (2HF highlighted in bright blue). Residues equivalent to A103 and V353 in *E. coli* (K103 and I342 in *T. maritima*; I94 and I356 in *M. jannaschii*) are shown as yellow spheres, with the distances between them (Cβ-Cβ) marked out: 43.5 Å in the open complex, and 34.1 Å in the closed complex. (**B**) FRET efficiencies of 100 nM SecY**EG in PLs (light grey) or DDM-solubilised (dark grey): with 1 µM SecA; with SecA and 1 mM AMPPNP; with SecA and 1 mM ADP; and alone. Data are normalised to SecYEG with SecA but without added nucleotide, and error bars represent the standard error from six repeats. **DOI:** http://dx.doi.org/10.7554/eLife.15598.008

The stability of the three conformations observed (O, PO and C, Figure 2—figure supplement 1) suggests that they represent genuine metastable states, however MD sampling is not sufficient to determine their relative populations. To test the MD results experimentally, donor (Alexa Fluor 488) and acceptor (Alexa Fluor 594) fluorescent probes were attached to a unique cysteine pair flanking the LG (A103 and V353 of *Escherichia coli* SecY; Figure 4A; Cβ-Cβ distances 3.4–4.4 nm; the Förster radius, R_0_, is 6 nm for the chosen dye pair). These allow the open and closed states of the LG to be distinguished by FRET. The doubly labelled protein (hereafter SecY**EG) is active (Figure 4—figure supplement 1A) and limited proteolysis confirms equal labelling on both sites (Figure 4—figure supplement 1B+C). After reconstitution into proteo-liposomes (PLs), about 50% (as judged by trypsin protection) of the complexes face outwards (Figure 4—figure supplement 1D), as has been observed previously for wild-type SecYEG (Mao et al., 2013; Schulze et al., 2014).

**Figure 4—figure supplement 1.**
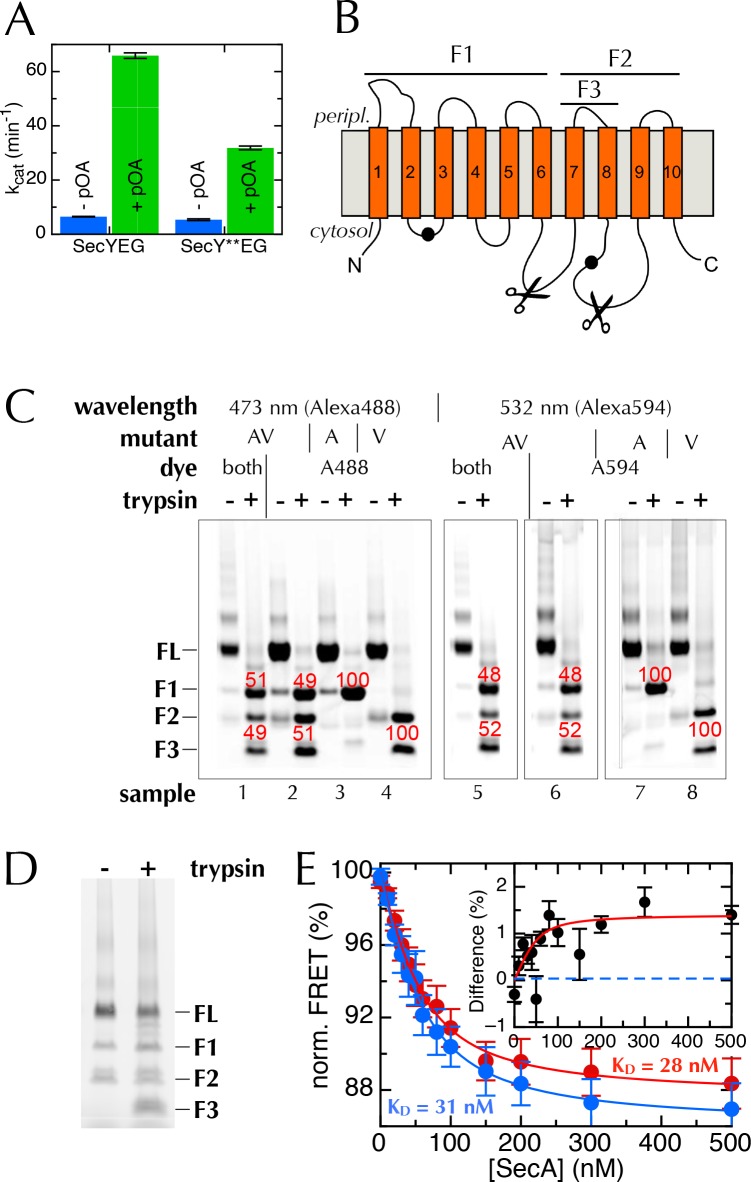
The extrinsic FRET pair of SecY**EG reports on the distance across the lateral gate. (**A**) ATPase assay on SecA in the presence of saturating amounts of SecYEG or SecY**EG PLs.Rates are shown both before and after addition of pre-protein (pOA) to 0.7 µM, and demonstrate that SecY**EG is active. (**B**) Schematic diagram of SecY, with the ten TMs shown, and the two labelling positions (A103 and V353) shown as black circles. The primary and secondary trypsin cleavage sites – between TMs 6–7 and TMs 8–9, respectively – are also marked out. Cleavage with trypsin yields one fragment of ~28 kDa containing the A103 position (F1), and another of ~21 kDa containing the V353 position (F2). F2 is partially cleaved to produce another 10 kDa fragment (F3), which also contains the V353 position. (**C**) Fluorescence visualisation of SDS-PAGE following trypsin cleavage of the two single mutants, SecYEG^A103C^ (A) and SecYEG^V353C^ (V), and the double mutant (AV) all conducted in DDM. Each pair of lanes shows SecYEG without (-) and with (+) trypsin treatment; in the absence of trypsin, fluorescent protein runs as a full length band (FL), while trypsin cleavage produces fragments F1–F3. Numbers in red show percentages of the total intensity for the bands in the lane. As expected, fluorophores on the A103 (A) position run in band F1 (samples 3 & 7), while fluorophores on V353 (V) are distributed between bands F2 and F3 (samples 4 & 8). Protein labelled with a single dye at both positions (samples 2 and 6) shows both positions labelled equally (samples 2 & 6). The SecY**EG mutant (samples 1 and 5) is labelled equally on both positions, with both dyes. Therefore, we can rule out an unexpected asymmetric dye loading on the two sites that would skew the FRET results. (**D**) Analysis of SecY**EG PLs by trypsin proteolysis, results shown without (-) and with (+) trypsin. Fluorescence quantification indicates that the band corresponding to full-length SecYEG accounts for 54% of the total signal in the '+' lane, once the lower molecular weight bands in the '-' lane are corrected for. This is equivalent to 46% outward facing SecYs: consistent, within error, with previous reports that show equal amounts of inward- and outward-facing SecYEG (Mao et al., 2013; Schulze et al., 2014). (**E**) Normalised FRET efficiencies of 50 nM SecY**EG in PLs in the presence of 500 µM ADP (red) or 500 µM AMPPNP (blue), as a function of SecA concentration. Data are fitted to a tight binding equation and show a difference in apparent FRET efficiency at saturation, but no measurable difference in affinity. Due to variability in protein preps, labelling efficiencies and reconstitutions, variation in the total magnitude of the signal was greater than the individual differences between ADP and AMPPNP. To account for this, we calculated the differences in FRET pairwise for each data set (inset). Error bars represent the standard error of mean (SEM) of five replicates, and the dotted blue line represents where the data would cluster if the ADP and AMPPNP titrations gave the same signal. **DOI:** http://dx.doi.org/10.7554/eLife.15598.009

**Figure 4—figure supplement 2.**
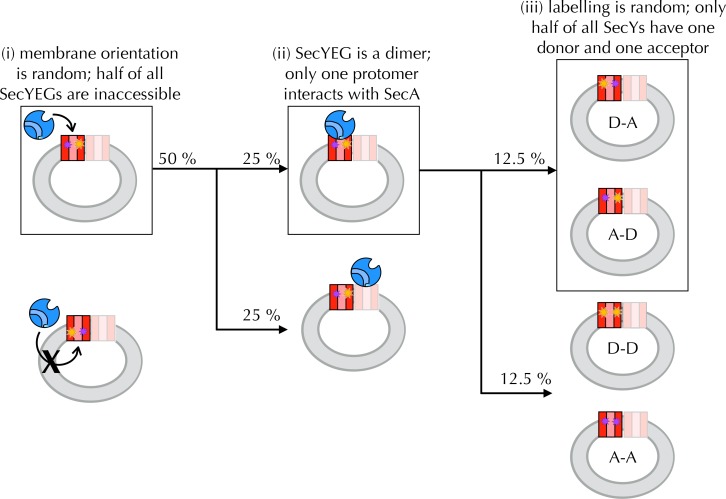
Interpreting ensemble FRET in PLs. The FRET changes observed in Figure 4B are lower than would be expected from the structures of the open and closed channel (Figure 4A), given the known R_0_ of the dye pair (6 nm). Three factors are likely to cause this: (i) about half of all SecY**EG molecules will be facing inwards after reconstitution (Mao et al., 2013; Schulze et al., 2014) (Figure 4S1) and will thus be unavailable for contact with SecA; (ii) SecYEG forms a dimer in the membrane (Bessonneau et al., 2002; Breyton et al., 2002) and SecA engages only one channel for translocation (Osborne and Rapoport, 2007; Zimmer et al., 2008; Deville et al., 2011); and (iii) at most half of the SecYs will carry the donor-acceptor pair due to the random labelling – possibly fewer if labelling is incomplete. In total, therefore, only ~12.5% of all SecYEG molecules are expected to respond to the presence of translocation partners. Two of these three problems are obviated by using single molecule FRET: (ii) is solved by using high lipid to protein ratio (see Methods for details) and extruding the PLs to 100 nm, such that only a single copy of SecY is present in each PL (Deville et al., 2011); and (iii) is solved because only particles containing a single donor and a single acceptor are used for analysis (see Methods and Figure 5—figure supplement 1). Because we use intact PLs, problem (i) still remains even at the single molecule level. We account for this in the final reckoning (Figure 5H) by assuming that half the particles do not respond to SecA as they are inward-facing (Figure 4—figure supplement 1D) and behave as SecA with no additional binding partners. This was done simply by removing 50% of the 'alone' populations from each other condition, then multiplying the result by two to give 100%. **DOI:** http://dx.doi.org/10.7554/eLife.15598.010

Equilibrium fluorescence measurements of SecY**EG show a decrease in FRET upon binding of SecA (Figure 4B), indicating LG movement – as expected from the crystal structures. A further FRET decrease is observed upon addition of the non-hydrolysable ATP analogue AMPPNP, but not ADP (Figure 4B). Titration of SecA into SecY**EG PLs in the presence of either ADP or AMPPNP (Figure 4—figure supplement 1E) shows that the changes in apparent FRET efficiency are not caused by differences in affinity between SecYEG and SecA in different nucleotide states, but rather represent differences in the width of the LG, *i.e.* a closure of the LG in the presence of ADP on SecA, some 5 nm away – consistent with the MD results.

### Single molecule analysis of the opening and closure of the lateral gate

Ensemble FRET measurements cannot be interpreted easily in terms of mechanistic details, because they report on the average of a mixed population of molecules. Only a fraction of SecYEG molecules are expected to be oriented correctly to engage substrate and labelled such that they give rise to a FRET signal (Figure 4—figure supplement 2), thus specific FRET changes can be drowned out in the ensemble signal.

**Figure 5.**
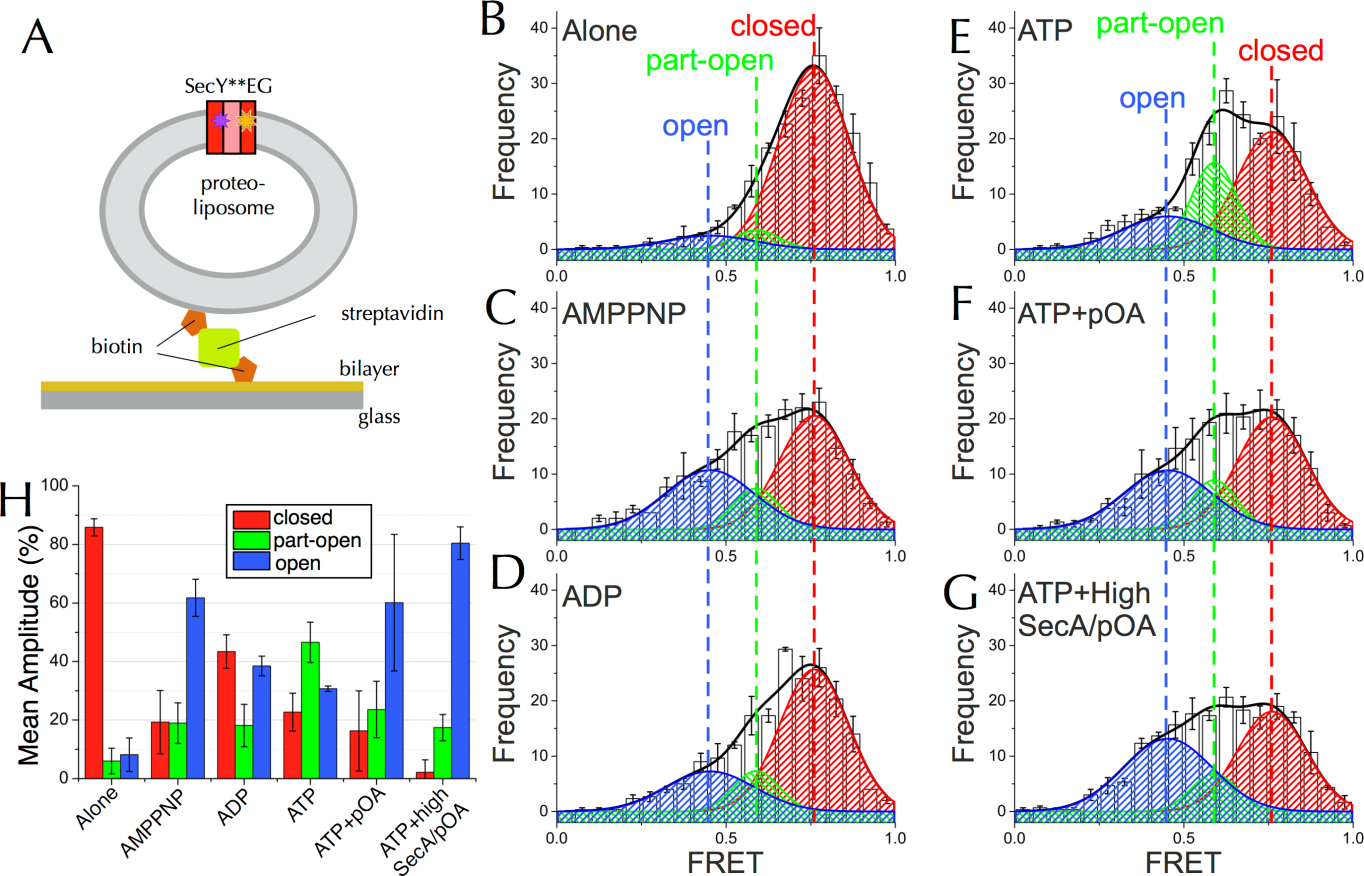
Communication from SecA to SecY: single molecule FRET analysis of lateral gate opening. (**A**) Schematic diagram of proteo-liposomes (PLs) containing a single SecY**EG complex used for single molecule TIRF experiments. PLs extruded to 100 nm were immobilised on the surface of a glass-supported lipid bilayer *via* a biotin-streptavidin-biotin bridge. (**B**–**G**) TIRF FRET efficiency distributions of SecY**EG PLs imaged alone (**B**) or incubated at room temperature for 15–40 min with 40 nM SecA in the presence of (**C**) 1 mM AMPPNP; (**D**) 1 mM ADP; (**E**) 1 mM ATP and an ATP regenerating system or (**F**) 1 mM ATP, 200 nM pOA and an ATP regenerating system, as well as (**G**) with 1 µM SecA, 1 mM ATP, 700 nM pOA and an ATP regenerating system. Each data set shows the average and SEM from three independent experiments (number of FRET events n = 200 each). Grey boxes represent histogram frequencies; red, green and blue shaded areas show least-squared Gaussian fits to high, medium and low FRET efficiencies, respectively. The black curve represents the sum of the fitted Gaussian distributions. (**H**) Amplitudes of the three TIRF FRET Gaussian peaks in panels (**B**–**G**). To correct for the inward-facing SecYEG molecules in the PLs (Figure 4—figure supplement 1D), 50% of the 'Alone' populations were subtracted from each sample (uncorrected data shown in Figure 5—figure supplement 2B). Bar heights are the mean from three replicates, and error bars represent the SEM computed by ANOVA analysis (full data shown in Figure 5—figure supplement 2A). **DOI:** http://dx.doi.org/10.7554/eLife.15598.011

To overcome this, we used single molecule FRET (smFRET). This technique allows the conformation of the LG of SecYEG to be monitored in the presence of SecA and different nucleotides, and can reveal the existence and proportions of different conformations within the ensemble. PLs containing single copies of SecY**EG were tethered to a glass supported bilayer as previously described (Figure 5A) (Deville et al., 2011). From time-lapse TIRF images, particles containing a single FRET pair as judged by sequential single step photobleaching of both dyes were selected for FRET analysis (see Materials and methods and Figure 5—figure supplement 1 for details). FRET efficiency was calculated for SecY**EG alone or with SecA and various nucleotides and plotted as histograms (Figure 5B-G and Figure 5—figure supplement 2A).

**Figure 5—figure supplement 1.**
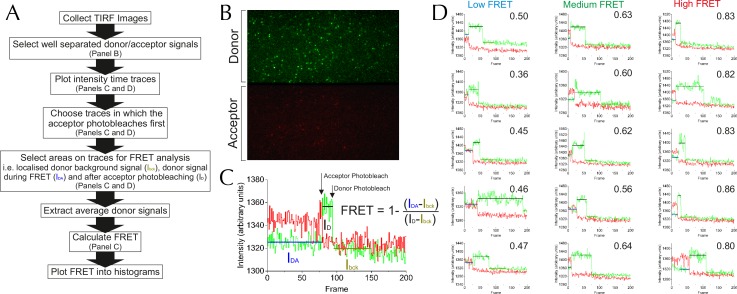
Methodology for TIRF analysis. (**A**) A flow diagram describing the process of analysing single molecule TIRF videos to generate FRET efficiency data (an example frame is displayed in Figure 5—figure supplement 1B). (**B**) TIRF image of immobilised SecY**EG illuminated by a 488 nm evanescent field. The upper panel displays the donor emission from AF488 (green) and the lower panel AF594 acceptor emission (red) across the same area. (**C**) Example of a single molecule SecY**EG TIRF fluorescence time trace (repetition time 0.22 s per frame, exposure time 0.2 s) used to calculate FRET efficiency, following the intensity of the donor (green) and acceptor (red) dye pair as a function of time. For data analysis, only traces with sequential single-step acceptor and donor photobleaching were used. Donor signals during FRET (I_DA_), and alone (after acceptor photobleaching, I_D_) corrected for background (signal after donor photobleaching, I_bck_) (right) were used to calculate FRET efficiencies according to the inset equation (Lakowicz, 2013). (**D**) Representative single molecule TIRF time traces of low, medium and high FRET efficiencies, labelled with their corresponding FRET efficiencies (calculated as in panel **C**). **DOI:** http://dx.doi.org/10.7554/eLife.15598.012

**Figure 5—figure supplement 2.**
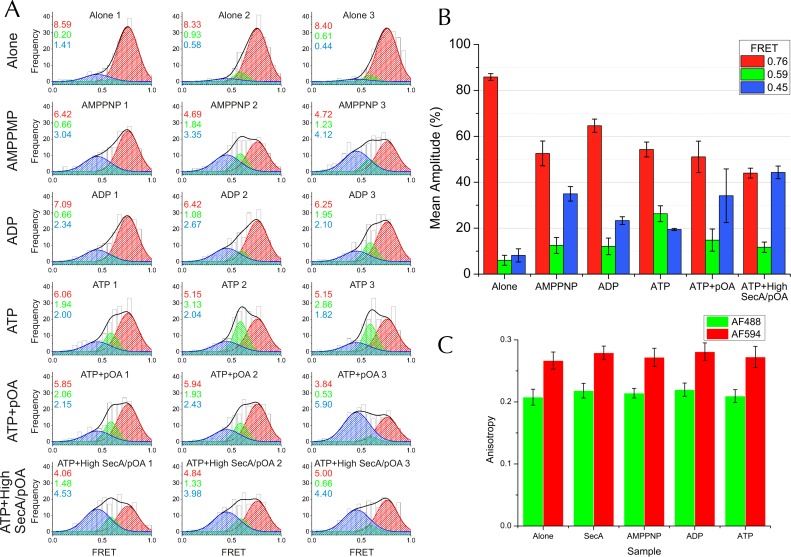
Complete single molecule FRET data. (**A**) TIRF FRET efficiency distribution of three independent repeats (n = 200 for each) for SecY**EG PLs imaged alone or incubated at room temperature for 15–40 min with 40 nM SecA and either 1 mM AMPPNP, 1 mM ADP, 1 mM ATP, or 1 mM ATP and 200 nM pOA (ATP+pOA), or with 1 μM SecA, 1 mM ATP and 700 nM pOA (ATP+High SecA/pOA). All samples with ATP also contained an ATP regenerating system (see Methods). Three Gaussian curves are required to describe all the data (see Figure 5—figure supplement 3A). To carry out the fitting, the parameters of the high FRET peak (position at E =0.76 and width of 0.24) were first estimated from a single Gaussian fit to the 'SecYEG alone' distributions. The complete data set was then fitted globally to three Gaussians, with the position and width of the high FRET peak fixed, and all other parameters (the amplitudes of all three peaks and the positions and widths of the remaining two peaks) were allowed to float. Next, the positions and widths of all three peaks were fixed while the amplitudes were unshared to allow variation between individual data sets. Finally, the positions and widths of the two lower FRET peaks were allowed to vary individually, along with all the amplitudes, to confirm that the model was not over-constrained. Black bars represent histogram frequencies; red Gaussians have a mean FRET of 0.76 (LG closed) with a width of 0.24; green Gaussians have a mean FRET of 0.59 (LG partially open) and width of 0.16; and blue Gaussians have a mean FRET of 0.45 (LG open) with a width of 0.31. Black curves represent the sum of the fitted Gaussian distributions. Amplitudes of the Gaussian fits are presented in the corresponding colour for LG FRET states in each plot. (**B**) Bar chart of amplitudes for the three FRET populations obtained by non-linear fitting, as in Figure 5H but prior to correction for inward-facing complexes. The averages and errors (SEM, standard error of mean) were obtained from the three independent data sets in panel **A** using one way ANOVA for each peak. (**C**) Fluorescence anisotropies for all the data sets. While calculating distances from FRET efficiencies may be affected by dye immobilisation and re-orientation, the observed anisotropies are constant between samples and indicate considerable dye mobility, thus permitting estimation of distance changes between states (Sharma et al., 2014). Note that no attempt was made to arrive at absolute distances between the dyes in each of the states, as this would require more rigorous consideration of dye attachment, movement and environment. **DOI:** http://dx.doi.org/10.7554/eLife.15598.013

**Figure 5—figure supplement 3.**
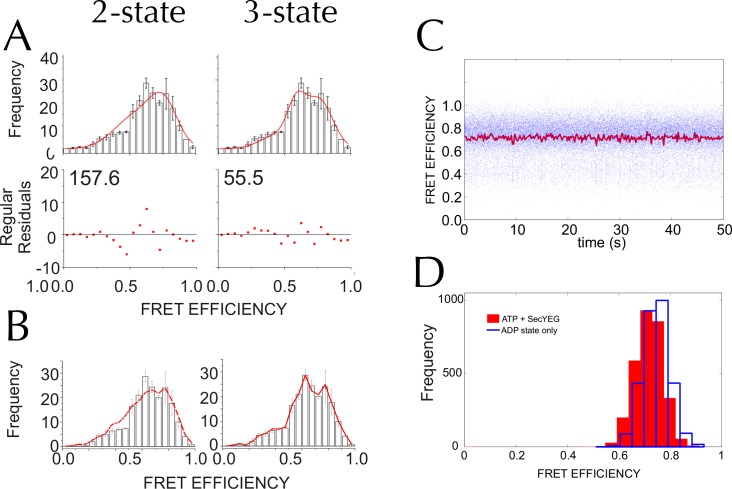
Three states are required to fit the smFRET data collected with ATP. (**A**) Comparison of two- (left) and three- (right) Gaussian fits to the TIRF FRET frequency distributions for the data in the presence of SecA and ATP (Figure 5E). Data show the average of three repeats for each condition with standard error bars, and the best non-linear global fit (averaged from the three individual fits) is shown as a red line. Residual plots are shown beneath each fitted distribution, along with their corresponding residual sum of squares value, as a measure of the quality of fit. Fits were carried out with position and width of the high FRET peak determined for the 'Alone' sample, then fixed for the remaining samples. (**B**) The whole set of histograms was subjected to SVD analysis and two (left) or three (right) most significant components were selected to describe FRET histogram (in the presence of ATP). As with the global non-linear fitting, three components are required to describe the ‘ATP' data to within experimental error. (**C**) Interconversion between the closed and open states on a timescale faster than our TIRF data collection (0.2 s per frame) could, in principle, give rise to an apparent third FRET peak due purely to averaging. In order to evaluate whether the intermediate peak (E = 0.59) corresponds to a real, part open state, or results from averaging of 2 distinct states during ATP hydrolysis, we simulated two-state traces (with FRET efficiencies of 0.76 and 0.45) with interconversion between the two states. Given that ADP release is the rate-limiting step in SecA, we set the dwell time in the high FRET (ADP bound, closed) state to 1/*k*_cat_ (measured previously [Robson et al., 2009]); while the dwell time in the low FRET (ATP bound, open) was set 1/*k*_cleave_, where *k*_cleave_ is the rate of ATP hydrolysis per se (11.5 s^-1^, as determined previously [Robson et al., 2009]). 50 s of simulated FRET efficiency time trace for SecYEG-SecA complex + ATP (*k*_cat_ = 0.27 s^-1^) are shown. Blue dots show the simulated raw data (with 1 ms time resolution), and the red line shows resulting simulated TIRF signal (averaged over 200 ms). (**D**) Histograms of FRET efficiency derived from the kinetic simulations, as in panel **C** (600 s total time for each). Two different conditions are shown: the ADP-bound closed state (blue, empty bars), and ATP turnover by SecYEG-SecA alone (red filled bars; as in panel **C**). The ADP-bound histogram is in good agreement with the experimental data (compare blue bars with Figure 5D). However, simulated ATP turnover does not recapitulate the experimental data (compare red bars with panel **A**): only a slight shift is seen of the high FRET peak, and no intermediate FRET feature appears. This suggests that in the case of slow ATP turnover, the intermediate FRET peak cannot be due to averaging and thus represents a genuine state. **DOI:** http://dx.doi.org/10.7554/eLife.15598.014

In the absence of SecA or nucleotide, SecY**EG exists mainly as a single population with an average FRET efficiency (E_FRET_) of 0.76 (Figure 5B). Such high efficiency is expected for the closed state structure (Figure 4A). In accord with the ensemble data, addition of SecA causes the open states to become more populated, with the relative populations dependent on which nucleotide is provided (Figure 5C–G). While a two state model could fit the majority of the distributions (data not shown) both global non-linear fitting (Figure 5—figure supplement 3A) and singular value decomposition (Figure 5—figure supplement 3B) indicate that at least three components are required to fully describe the histogram distribution with ATP. Therefore three principal states were used to fit the entire dataset: (1) closed, E_FRET_ ~0.76 (red); (2) part-open, E_FRET_ ~0.59 (green); and (3) open, E_FRET_ ~0.45 (blue).

Estimating absolute distances from E_FRET_ values is not warranted in such a complex system, particularly as the dyes are attached by long, flexible linkers. However, anisotropy measurements indicate that the dyes retain rotational freedom in all states (Figure 5—figure supplement 2C). Hence, relative movements can be approximated from changes in E_FRET_. Assuming an R_0_ of 6 nm, the difference between the open and closed state is ~0.9 nm – consistent with the changes observed across LG by MD (Figure 2C) and those expected from the crystal structures (Figure 4A).

After adjusting for ~50% of SecY**EG oriented such that it cannot bind SecA (Figure 4—figure supplements 1 and 2) the relative percentages of closed, open and part-open can be determined for active SecYEG. These data (Figure 5H) show that in the presence of AMPPNP – which traps the complex in an ATP bound-like state—the LG occupies mainly the open conformation (Figure 5C+H), while with ADP the closed state is more highly populated (Figure 5D+H). Intriguingly, in the presence of ATP—which under steady-state conditions is rapidly hydrolysed to ADP (Robson et al., 2009)—SecY predominantly populates the part-open state (Figure 5E+H). Mathematical modelling indicates that the third state cannot be explained by averaging of the two different nucleotide bound forms during data collection (Figure 5—figure supplement 3C+D). So, the part-open state most likely represents a true intermediate on the pathway between the closed and open conformations.

Addition of pOA + ATP to SecY**EG-SecA causes the open state to dominate (Figure 5F+H); an effect consolidated by addition of a large excess of SecA and pOA (Figure 5G+H). This is consistent with the known properties of the steady-state cycle of SecA: ADP release is strongly accelerated by the presence of pre-protein, such that the open (ATP-bound) state becomes more highly populated (Robson et al., 2009). No further states of the channel are detected in the presence of the pre-protein (Figure 5F+G). Taken together, the MD and FRET results show that nucleotide occupancy in SecA regulates the opening of the SecY protein-channel and LG.

### Closure of the SecY lateral gate alters ATP turnover by SecA

**Figure 6.**
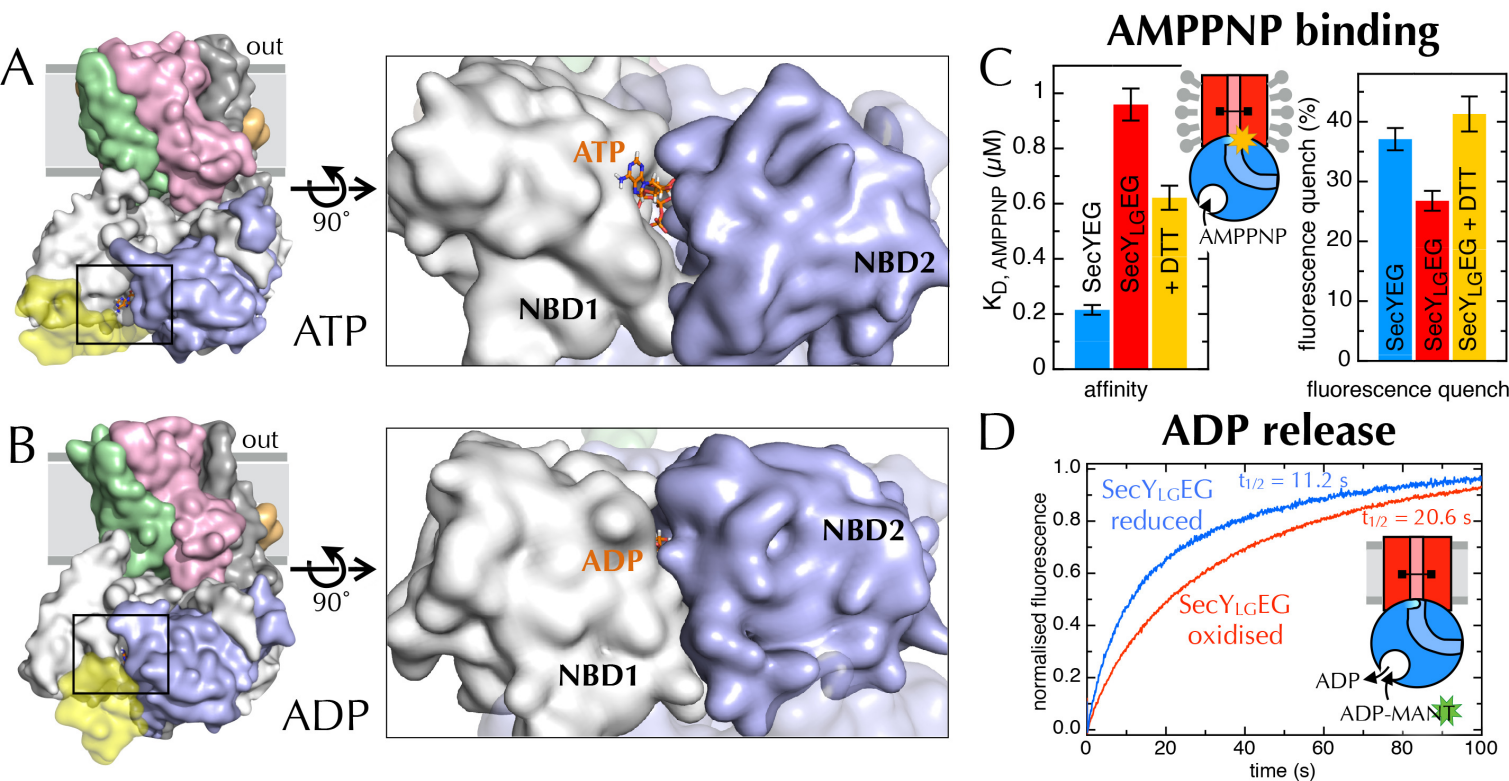
Communication from SecY to SecA: lateral gate-dependent changes in the NBS of SecA. (**A**) Surface view of SecYEG-SecA after 1 µs MD with ATP. SecY is coloured pink, SecE orange, SecG green and SecA white, with NBD2 highlighted in blue, and the domain exclusively found in thermophilic organisms coloured yellow. ATP is shown as sticks and coloured orange, with nitrogens blue, oxygens red and hydrogens white. In the closeup of the NBS (right), the yellow loop on SecA is omitted for clarity. (**B**) Same as panel **A**, but after MD with ADP. (**C**) Affinity for AMPPNP (left) and maximum fluorescence quenching (right) for SecA* in the presence of SecYEG (blue), SecY_LG_EG (red), or SecY_LG_EG with 100 mM DTT (yellow). AMPPNP was titrated into 200 nM SecA* in the presence of saturating (1 µM) SecYEG and fitted to the tight binding equation (see methods); error bars represent the SEM of three repeats. Note that 100 mM DTT is enough to fully reduce the disulphide bond (Figure 6—figure supplement 2C), and restore the fluorescence amplitude; the incomplete restoration of AMPPNP affinity is therefore most likely an effect of the cysteine substitutions. Note also a minor reduction in the affinity of SecY_LG_EG for SecA in detergent (Figure 6—figure supplement 3A)—normally characteristic of the ADP-bound state (Deville et al., 2011). (**D**) Stopped flow fluorescence time courses of ADP release, after equilibrating 0.6 µM SecA and 1 mM ADP with either 2.4 µM SecYEG (blue) or SecY_LG_EG (red) in PLs. Time courses were measured by rapidly mixing with 25 µM MANT-ADP and following fluorescence with excitation at 296 nm and emission measured using a 399 nm longpass filter. Data were fitted to double exponentials and normalised to give a total amplitude of 1. **DOI:** http://dx.doi.org/10.7554/eLife.15598.015

In addition to the LG movement, the MD simulations showed differences in the NBS of SecA, which turns out to be more open in the ATP simulations compared to those done with ADP bound (Figure 6A+B). This can be quantified by calculating the nucleotide solvent accessible surface (Figure 6—figure supplement 1A; calculated with the GROMACS utility g_sas [Eisenberg and McLachlan, 1986]). It seems likely that an open NBS would increase the rate of nucleotide exchange, altering its affinity and turnover.

**Figure 6—figure supplement 1.**
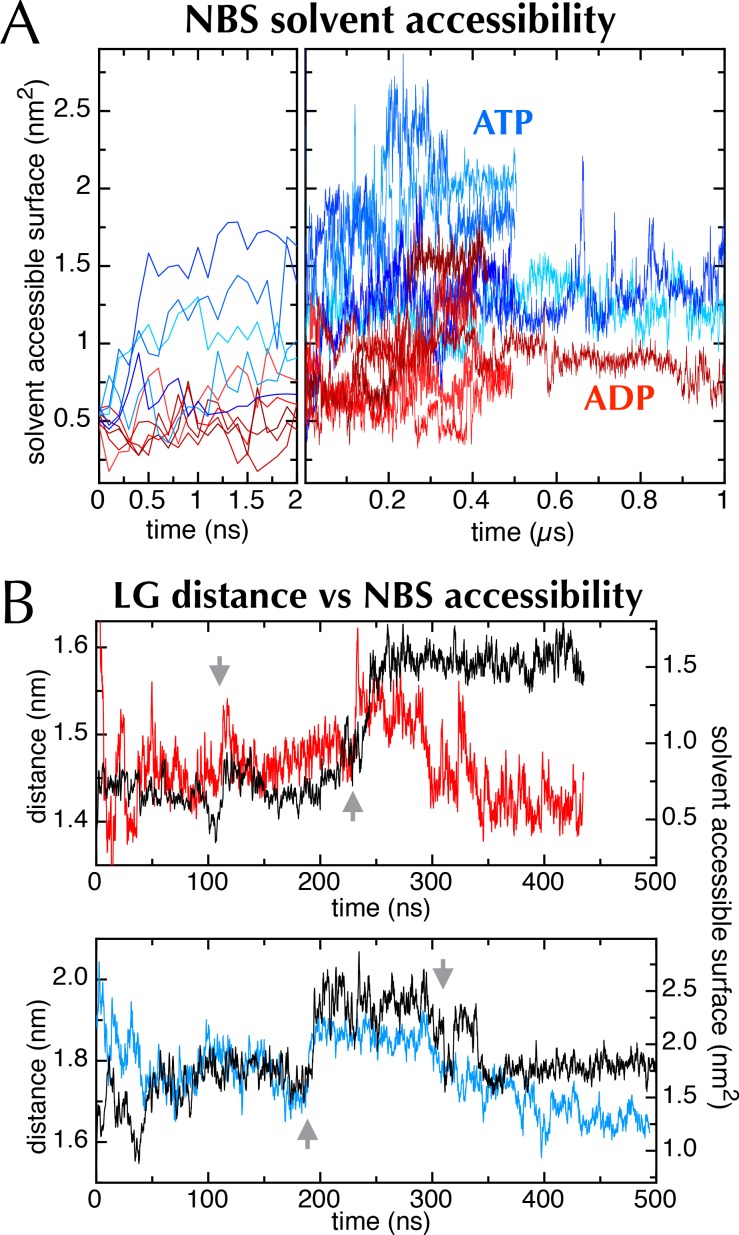
The nucleotide binding site in MD simulations. (**A**) Nucleotide solvent accessible surface (calculated with the GROMACS utility g_sas (Eisenberg and McLachlan, 1986) and adjusted for size of the nucleotide) as a function of simulation time for all 10 MD traces shown in Figure 2C. Some of the ATP and ADP samples diverge within the first ns; to show this, the first 2 ns of the plot is expanded. The ATP-containing samples generally occupy more open conformations, as shown in Figure 6A–B. (**B**) Comparison of distance across the SecY LG (red line for ADP (upper panel), blue line for ATP [lower panel]) with SecA nucleotide binding site solvent accessible surface (black lines [both panels]). Both MD simulations exhibit sudden changes in distance across the LG, which coincide with abrupt changes in the NBS (grey arrows), lending qualitative support to the idea that the two distal sites are communicating. **DOI:** http://dx.doi.org/10.7554/eLife.15598.016

**Figure 6—figure supplement 2.**
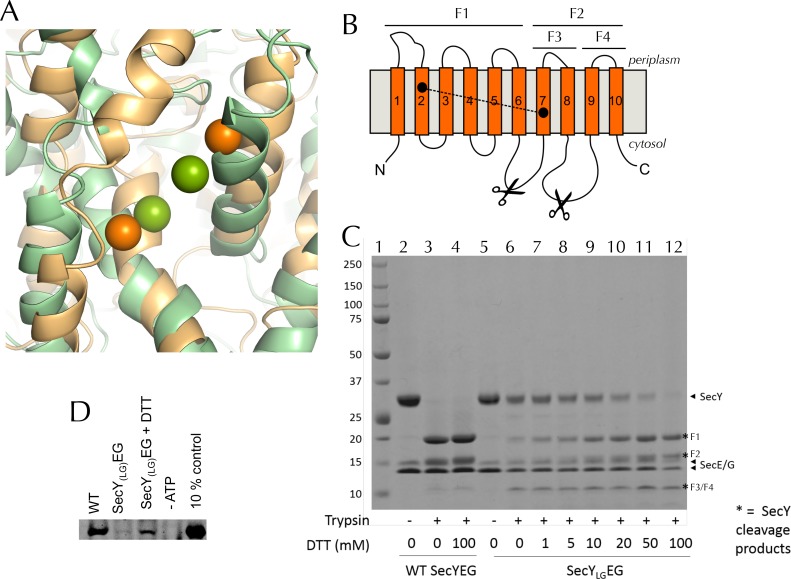
Validation of SecY_LG_EG (**A**) Post-100 ns MD snapshots of the LG of uncrosslinked SecY_LG_EG-SecA with ADP (green) and ATP (orange). The γ-sulphur atom of each cysteine is shown as a sphere. The simulations demonstrate that the double cysteine mutation used to crosslink the LG of SecY does not prevent the nucleotide-dependent conformational changes observed in the wild-type complex (Figure 2C). (**B**) Schematic diagram of SecY, with the ten TMs labelled and the SecY_LG_ crosslinking sites (S87C and F286C) shown as black circles. The primary and secondary trypsin cleavage sites – between TMs 6–7 and TMs 8–9, respectively – are also indicated. Cleavage with trypsin yields two major fragments (F1 and F2), with F2 partially cleaved again (into F3 and F4). Note that if a disulphide bond is formed (dashed line), F1/F2 and F1/F3 will not separate by non-reducing SDS-PAGE and will resemble full length SecY. (**C**) Coomassie stained SDS-PAGE of a trypsin proteolysis experiment, to test the degree of crosslinking of DDM-solubilised SecY_LG_EG. Full-length SecY runs at ~32 kDa, and the primary SecY trypsin fragments at ~19 and 15 kDa. The first lane (1) contains a molecular weight ladder. Other lanes, from left to right, contain: wild-type SecYEG, untreated (2); treated with 0.75 µg.ml^-1^ porcine trypsin (3); or treated with trypsin after incubation with 100 mM DTT (4). Lanes 5–12 contain SecY_LG_EG alone (5) or treated with trypsin following incubation with various concentrations of DTT as indicated in the panel. The full-length wild-type SecY is fully cleaved both with and without DTT. In contrast, the oxidised SecY_LG_EG (no DTT) digest resembles that of the intact (no trypsin) wild-type SecY, and is progressively more cleaved with increasing DTT concentration, until it is almost fully cleaved at 100 mM DTT. The results are consistent with essentially complete disulphide bond formation. Note that fragment F4 runs at ~12 kDa. As its cleavage site is unaffected by the disulphide, it is present in the same amount in all lanes that have been treated with trypsin. Note also that reconstitution into PLs occludes the trypsin cleavage site of inward-facing SecYs, so the degree of crosslinking for SecY_LG_EG cannot be determined in a lipid environment. (**D**) Representative blot showing translocation of pre-protein by SecY_LG_EG driven into the interior of PLs by SecA and ATP. Reaction lanes show a western blot of the protease-protected pOA after a 30 min translocation reaction. The -ATP lane is a negative control, using the same components but lacking ATP. The 10% lanes show the starting material, untreated with protease K (1/10th the amount loaded). As reported previously (du Plessis et al., 2009) the SecY_LG_EG mutant is severely compromised in translocation, but activity is partially restored by addition of DTT. **DOI:** http://dx.doi.org/10.7554/eLife.15598.017

**Figure 6—figure supplement 3.**
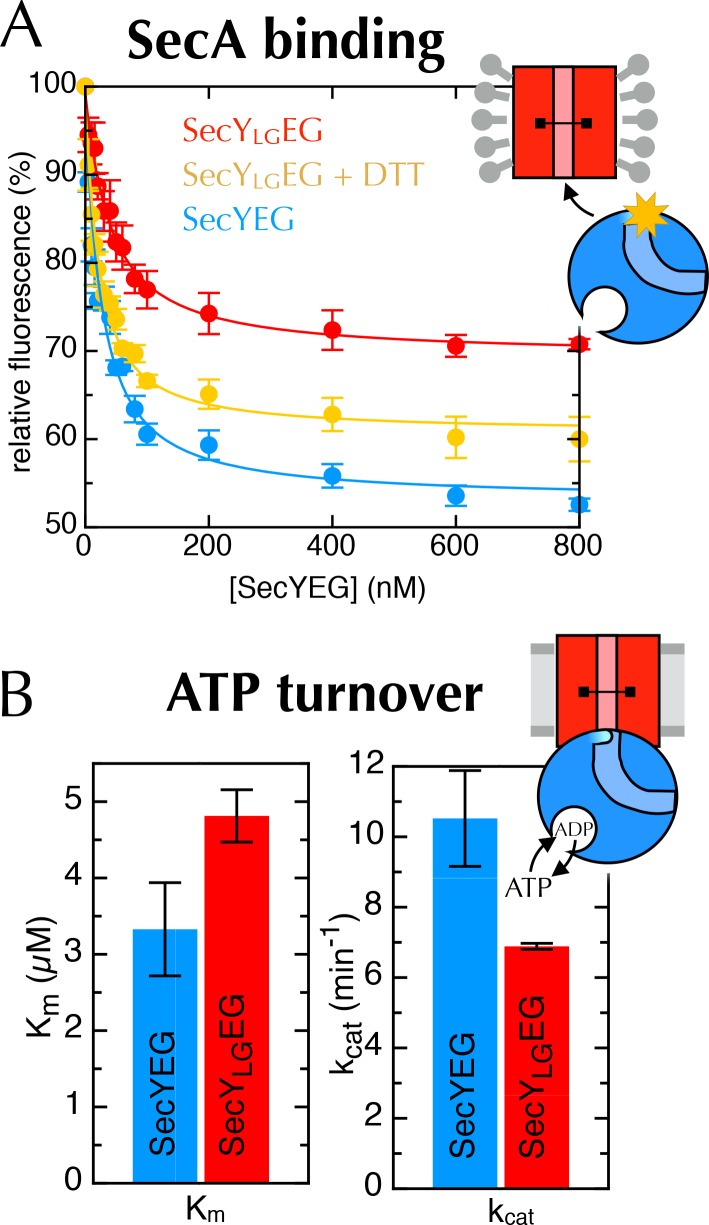
Additional data for the crosslinked SecY_LG_EG. (**A**) Fluorescence of 10 nM SecA* upon titration with SecYEG (blue), SecY_LG_EG (red), or SecY_LG_EG pre-incubated with 100 mM DTT (yellow), in the presence of 1 mM AMPPNP and normalised to SecA* alone. Error bars represent the SEM from three repeats, and data were fitted to the tight binding equation, yielding K_D_ = 23.5 ± 0.2 nM, B_max_ = 47 ± 1% for SecYEG; K_D_ = 41 ± 7 nM, B_max_ = 31 ± 1% for SecY_LG_EG; and K_D_ = 19 ± 3 nM, B_max_ = 40 ± 1% for SecY_LG_EG + DTT. As can be seen, crosslinking the LG causes a minor (<2-fold) reduction in affinity for SecA, but a more striking difference in B_max_ (as seen also in Figure 6C). (**B**) Steady-state ATPase kinetic parameters for SecA in the presence of SecYEG (blue) or SecY_LG_EG (red). Bar heights are average fits to the Michaelis-Menten equation for three repeats, and error bars represent the SEM. The raised K_m_ and lowered *k*_cat_ are consistent with both ATP binding and ADP release being compromised upon cross-linking the LG—*i.e.* closing of the NBS. This also supports the results shown in Figure 6C+D. **DOI:** http://dx.doi.org/10.7554/eLife.15598.018

A detailed examination of the MD data hints at a correlation between the width across the LG and accessibility of the NBS (Figure 6—figure supplement 1B), which suggests a more fundamental coupling between the two distant sites. If this communication is two-way—*i.e.* changes in SecY cause the NBS to open or close—it could provide a mechanism of coupling protein translocation to nucleotide exchange. To explore this possibility, a cysteine pair was introduced across the LG such that disulphide bond formation traps the translocon in the closed state (du Plessis et al., 2009) (SecY_LG_EG; Figure 6—figure supplement 2A). Analysis of this sample by trypsin cleavage using SDS-PAGE shows >95% cross-linking (Figure 6—figure supplement 2B+C), and as reported previously (du Plessis et al., 2009) the trapped complex is incapable of protein translocation (Figure 6—figure supplement 2D).

Previously, we have shown that the fluorescence of a fluorescein label on the tip of the 2HF (position 795 of *E. coli* SecA; hereafter SecA*) is strongly quenched upon association with SecYEG, but only in the ATP (AMPPNP) bound state, and not with ADP (Deville et al., 2011). We exploited this by titrating AMPPNP into SecYEG-SecA* or SecY_LG_EG-SecA* to measure their relative affinities. Trapping the LG in a closed position causes a 5-fold reduction in affinity of SecA for AMPPNP (Figure 6C, left), demonstrating that the conformational state of the LG is transmitted to the NBS. The degree of quenching is also reduced, from 36% to 26% (Figure 6C, right and Figure 6—figure supplement 3A), hinting that the channel entrance occupies a more 'ADP-like' conformation.

The rate-limiting step during ATP turnover is the dissociation of ADP (Robson et al., 2009), so we also examined the effects of LG closure on the rate of ADP release from SecA (*k*_off_). This can be measured by following competitive binding of a fluorescent nucleotide analogue (MANT-ADP) in a stopped flow apparatus (Robson et al., 2009). We found that the *k*_off_ is slowed by ~50% with a cross-linked LG (Figure 6D), consistent with the results presented above showing that NBS and LG closure are coordinated. As expected, this cross-talk is also reflected in the steady-state ATP turnover parameters (Figure 6—figure supplement 3B). Together, the results establish the existence of a two-way communication between SecYEG and SecA over a distance of >5 nm: SecA ATP binding and hydrolysis regulate the aperture of the channel, while LG opening and closing controls the ATP affinity and rate of ADP release from SecA. Interestingly, binding of signal sequence into the LG is known to be an allosteric activator of SecA (Gouridis et al., 2009; Hizlan et al., 2012): the increase in the turnover of ATP caused by LG opening could provide a basis for this effect.

### Substrates are sensed at the entrance of the protein channel – a role for the two-helix finger?

**Figure 7.**
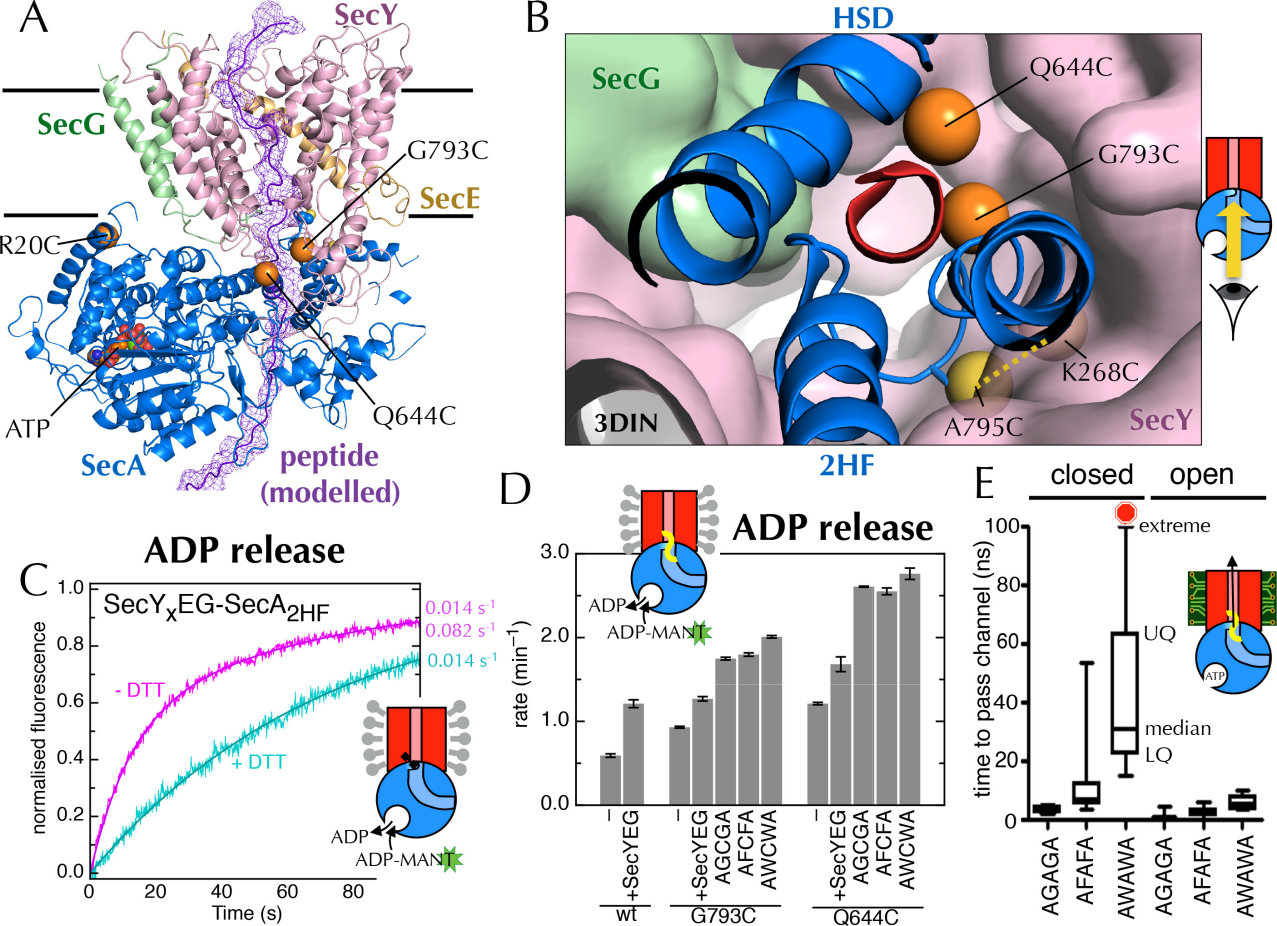
Involvement of the 2-helix finger in SecY <–> SecA communication. (**A**) Overview cartoon structure of the 1 µs open SecYEG-SecA complex with SecY in pink, SecE orange, SecG green and SecA blue. Residues 27–76 of *E. coli* pOA, shown as a purple ribbon with a mesh surface, have been modelled into a likely pathway through the channel, based on known crosslinking data through SecA (Bauer and Rapoport, 2009) and SecY (Cannon et al., 2005). The three peptide crosslinking sites on SecA (R20C, Q644C and G793C in *E. coli* numbering; K32C, T640C and S780C in *T. maritima*) are shown with the Sγ atoms as orange spheres. (**B**) Closeup structure of the SecY channel entrance with SecA bound (*T. maritima*, PDB code 3DIN [Zimmer et al., 2008]), looking into the channel from the cytosolic side and coloured as in panel A. SecY, SecE and SecG are shown as surfaces, while the 2HF and HSD (helical scaffold domain) of SecA are shown in cartoon representation. The SecA peptide cross-linking sites SecA^644^ and SecA^793^ in *E. coli* (respectively SecA^640^ and SecA^780^ in *T. maritima*) are indicated by orange spheres; while the ATPase-activating SecA-SecY cross-linking sites (Whitehouse et al., 2012) (residues SecA^795^/SecY^268^ in *E. coli*, SecA^782^/SecY^264^ in *T. maritima*) are yellow spheres. To give an impression of size a short α-helix (red) has been modelled into the channel opening. (**C**) Stopped flow fluorescence traces of ADP release by 0.6 µM SecY_x_EG-SecA_2HF_ and 1 mM ADP without (magenta) or with (turquoise) 30 min pre-incubation with 100 mM DTT. Data were fitted to a double (magenta) or single (turquoise) exponential as described in methods. (**D**) ADP release rates determined by fluorescence stopped flow for wild-type SecA (wt), SecA^G793C^ and SecA^Q644C^. Rates are shown for SecA alone; with an excess of SecYEG; and, where relevant, crosslinked to the peptides AGCGA, AFCFA or AWCWA (and in the presence of saturating SecYEG). Error bars represent the SEM from three independent experiments. Stopped flow traces are shown in Figure 7—figure supplement 2B. (**E**) Transit time for the peptides AGAGA, AFAFA or AWAWA through the closed (left) and open (right) SecY pore during constant force steered MD simulations. Each simulation was repeated ten times to produce the box plots. Two AWAWA peptides failed to cross the closed SecY channel within 100 ns (red octagon). **DOI:** http://dx.doi.org/10.7554/eLife.15598.019

The 2HF of SecA is situated directly on the path of pre-proteins as they enter the SecY-channel (Figures 1B and 7A+B), and we have previously shown that perturbing the 2HF by cross-linking to SecY (A795C of SecA cross-linked to K268C of SecY; hereafter SecY_x_EG-SecA_2HF_; yellow dotted line in Figure 7B) stimulates the ATPase activity of SecA ~25-fold in PLs (Whitehouse et al., 2012). At the time, this observation was surprising; however, in light of the results above, we hypothesised that the 2HF might be the sensor that couples the movements of the LG and the NBS. Indeed, the 2HF is known to play a crucial role in coupling ATP hydrolysis to translocation (Erlandson et al., 2008).

**Figure 7—figure supplement 1.**
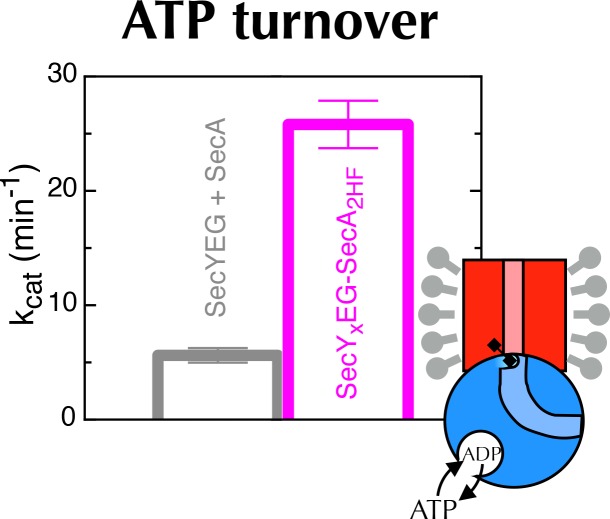
ATP turnover by crosslinked SecY_x_EG-SecA_2HF._ Turnover of ATP by SecYEG-SecA (SecA in the presence of saturating SecYEG; grey) or SecY_x_EG-SecA_2HF_ (magenta) in detergent solution. Error bars represent the SEM from three repeats. **DOI:** http://dx.doi.org/10.7554/eLife.15598.020

**Figure 7—figure supplement 2.**
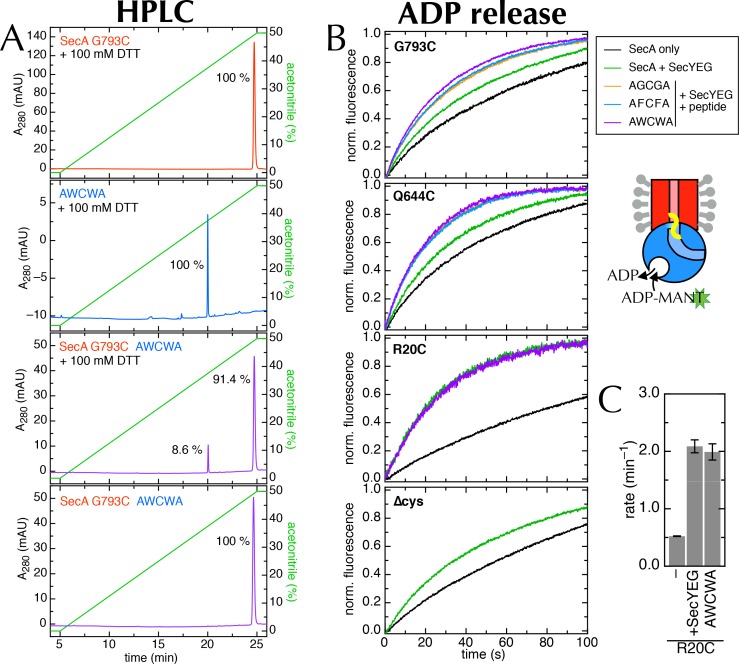
Supporting data for the 2HF labelling. (**A**) HPLC traces of SecA^G793C^ (Figure 7A), the peptide AWCWA, and SecA^G793C^ crosslinked to AWCWA, either with or without 20 min pre-incubation 100 mM DTT to break the disulphide bond. HPLC with DTT confirms that AWCWA is present on the crosslinked protein, while HPLC without DTT confirms that they are indeed connected by a disulphide bond. Dividing the peak integrals by the extinction coefficients of the components (ε = 99,000 M^-1^.cm^-1^ for SecA, determined experimentally; and ε = 11,380 M^-1^.cm^-1^ for AWCWA, calculated) gives an estimated labelling efficiency of 82%. Note that incomplete reduction of the disulphide bond would lead us to underestimate this value. Because only the tryptophan-containing (AWCWA) peptide has an appreciable UV absorption signal, we were unable to quantify labelling with the other two peptides. However, as the peptide labelling experiments were performed in parallel, and AWCWA is the least soluble of the three, AGCGA and AFCFA are likely to have crosslinked with the same or better efficiencies. (**B**) Stopped flow time traces for the data shown in Figure 7D and panel **C**. For ease of comparison, data were fitted to a single exponential, then normalised to an amplitude of 1. Note that SecA^R20C^ was not tested with AGCGA or AFCFA, and SecA_∆cys_ was not tested with any peptide, as it has no cross-linking site. (**C**) Rates of ADP release for SecA^R20C^ alone; with excess SecYEG; and crosslinked to the peptide AWCWA (and in the presence of excess SecYEG). For this position, which is not near the entrance to SecY (Figure 7A), the crosslinked peptide has no effect on the rate of ADP release. **DOI:** http://dx.doi.org/10.7554/eLife.15598.021

**Figure 7—figure supplement 3.**
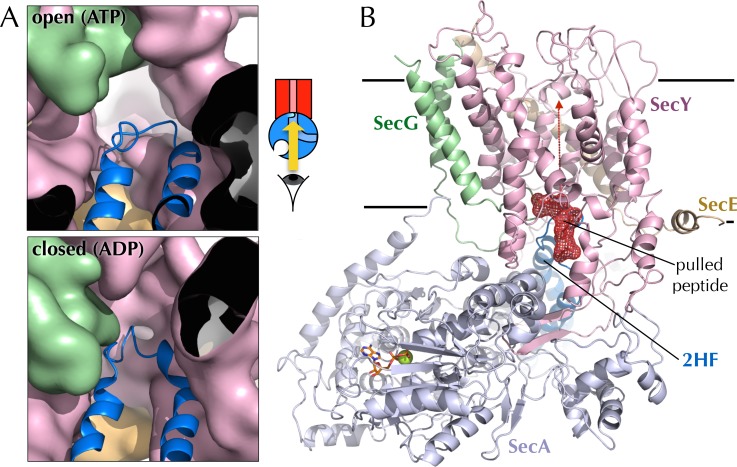
Entrance to the protein channel of SecY (**A**) Closeup of the SecY channel entrance after 1 µs MD in a representative open (ATP, above; O in Figure 2C) and closed (ADP, below, C in Figure 2C) conformations. SecYEG is shown as a surface with SecY in pink, SecE in orange and SecG in green, while the 2HF of SecA is shown as a blue cartoon. Note that LG closure favoured by ADP leads also to closure of the central protein channel. (**B**) Side-view of SecYEG-SecA with the pentapeptide AWAWA modelled at the interface between SecY and SecA. SecY, E, G and A are shown as pink, orange, green and pale blue cartoons, respectively. The 2HF is highlighted in bright blue, and the peptide is shown as red sticks overlaid with a red mesh. The direction of pulling in the steered MD experiments (Figure 7E) is indicated by a red dotted arrow. **DOI:** http://dx.doi.org/10.7554/eLife.15598.022

To probe the importance of the 2HF, we first confirmed that the increase in ATPase activity is also reflected in an increased *k*_off_ for ADP (using stopped flow as above; Figure 7C; ATPase increase in detergent shown in Figure 7—figure supplement 1). Next, to mimic a translocating pre-protein more directly, we introduced a cysteine at position 793 of *E. coli* SecA, *i.e.* on the 2HF (Figure 7A+B, orange sphere) and cross-linked a range of penta-peptides to it. The peptides were chosen to have different sequences (containing small or bulky side-chains) with a central cysteine for disulphide bond formation to the single engineered cysteine on SecA. A high degree of cross-linking was confirmed for the most hydrophobic substrate, AWCWA, using reverse-phase HPLC in the presence or absence of DTT (Figure 7—figure supplement 2A).

While the cysteine cross-linking site of SecA itself has no effect on the SecYEG stimulated release of ADP (Figure 7D, compare 'wt + SecYEG ' to 'G793C + SecYEG'), covalently cross-linked peptides elicit a significant increase in ADP *k*_off_ (Figure 7D and Figure 7—figure supplement 2B). The bulkier peptide, AWCWA, has a greater effect than peptides with smaller residues (AGCGA or AFCFA). A similar effect was observed when the same peptides were cross-linked to the opposite side of the putative peptide binding path (on the helical scaffold domain (HSD); position 644 of *E. coli* SecA; Figure 7A,B+D and Figure 7—figure supplement 2B), but not at an unrelated position (position 20 of *E. coli* SecA; Figure 7A and Figure 7—figure supplement 2B+C).

Thus, we conclude that peptides are sensed at the SecY channel entrance, and that this information is transmitted back to the NBS. As both pulling the 2HF away from the channel (with a disulphide bond) and pushing it away (with a bulky peptide; Figure 7B) elicit an increase in ADP release, it seems plausible that the 2HF is sensing the passing peptide sterically; the dramatic effects of point mutations in the 2HF (Erlandson et al., 2008; Bauer et al., 2014) are consistent with this interpretation.

### The closed channel poses a barrier to the passage of bulkier regions of substrate

Previous studies of translocation by SecYEG-SecA have shown that the ease with which a stretch of polypeptide chain passes through the substrate channel depends on the nature of the amino acids in that stretch (Sato et al., 1997; Bauer et al., 2014). For example, poly-glycine regions diffuse freely and rapidly backwards and forth (Bauer et al., 2014), while charged amino acids, especially positively charged, are excluded (Nouwen et al., 2009; Liang et al., 2012). It has also been shown that some combinations of amino acids form secondary structural elements even before they exit the ribosome (Hardesty and Kramer, 2001; Lu and Deutsch, 2005), while others remain natively unfolded. Given that the aperture of the SecY channel is nucleotide-dependent, we thought it might form a choke point for diffusion of bulky or structured regions of a substrate sequence: in the ADP-bound state, only uncharged, unfolded stretches of polypeptide may pass; whereas the ATP-bound state is less restrictive (Figure 7—figure supplement 3A).

To test this idea, we carried out steered MD experiments. Short peptides corresponding to those used for the cross-linking experiments (above) were modelled into the post-simulation ‘closed’ and ‘open’ structures, at the SecY channel entrance (Figure 7—figure supplement 3B), and pulled though the channel with a constant directional force. As expected given the dimensions of the open channel (Figure 7—figure supplement 3A), all three substrates crossed without difficulty (Figure 7E). For the closed structure however, while AGAGA readily passes through the channel, the larger substrates – especially AWAWA – are substantially retarded (Figure 7E). This indicates that the closed conformation – favoured by ADP occupied SecA – can permit passage of narrow regions of substrate, but selects against more bulky stretches. To allow these bulky regions through, the channel would need to open – as occurs when ATP is bound.

## Discussion

Over a decade has passed since the structure of the SecY complex was first determined (van den Berg et al., 2004). However the mechanism of translocation—how ATP turnover by SecA drives unfolded pre-proteins across the membrane through SecY—is not yet understood. At present, the most favoured model posits that substrate is pushed through the channel by the 2HF, with ATP providing energy *via* a power stroke (Erlandson et al., 2008). This model has recently been updated to allow free diffusion of some stretches of less bulky residues (Bauer et al., 2014); however, as we have argued previously (Collinson et al., 2015), evidence for the power stroke itself is lacking. Indeed, it is not clear from the structure of SecYEG-SecA where the 2HF would move, and how it could selectively bind and move both hydrophobic and charged regions of substrate. Furthermore, as immobilisation of the 2HF inside the channel does not prevent translocation (Whitehouse et al., 2012), any 'power-stroke' conformational change would have to be extremely subtle.

Here, we have combined all-atom molecular dynamics simulations with single molecule FRET and ensemble biochemical assays to investigate the events that couple the ATPase cycle of SecA with the rest of the complex. Four key observations were made: (i) the SecY channel is predominantly open with ATP bound to SecA and closed with ADP; (ii) the aperture of the channel regulates nucleotide exchange in SecA; (iii) this regulation is sensitive to the size of substrates at the SecYEG channel entrance; and (iv) the closed channel provides a selective barrier against larger substrates.

**Figure 8.**
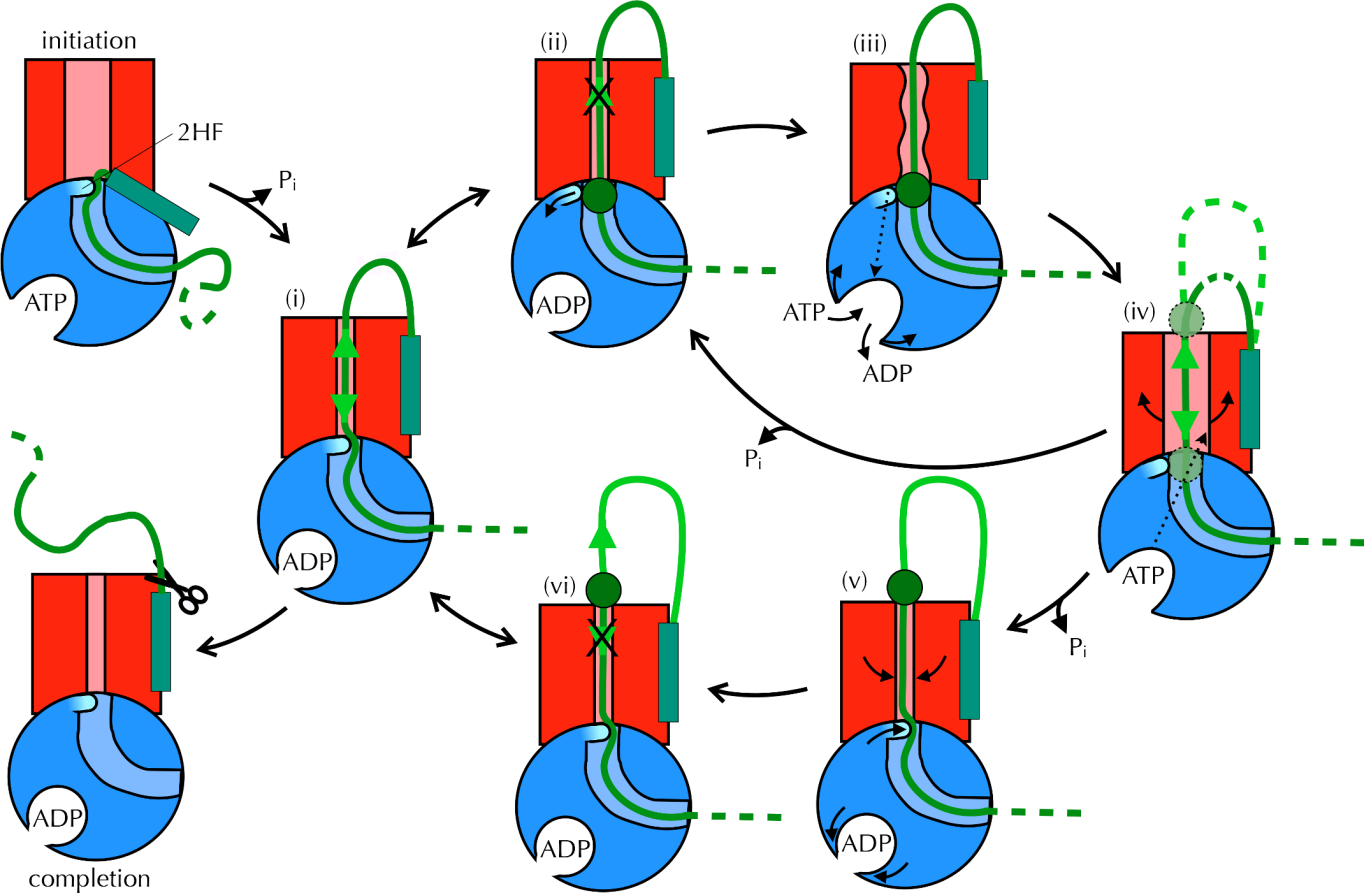
Proposed Brownian ratchet mechanism for translocation. SecYEG is shown in red (LG in light red), SecA in blue (substrate channel in light blue and 2HF in cyan and indicated), and substrate in green (with signal sequence as a turquoise rectangle). The initiation process (top left) involves conformational changes that prime the SecY-complex and intercalate the pre-protein (Hizlan et al., 2012; Corey et al., 2016). Subsequently, the substrate is free to diffuse backwards or forwards (i) until it reaches a block (green circle) at the entrance (ii) or exit (vi) to the channel. A block at the entrance triggers nucleotide exchange (iii), which leads to opening of the LG (iv). The wider channel permits diffusion of the blocked region of substrate within the pore, until ATP hydrolysis recloses the channel, trapping the block either outside (v) or inside (ii) the membrane. This cycle produces a net forwards driving force because blocks at the channel exit (vi) do not trigger nucleotide exchange and channel opening; the substrate is therefore ratcheted in one direction. Once the entire chain emerges from the channel it can no longer diffuse backwards and cleavage of the signal sequence is all that remains (completion). **DOI:** http://dx.doi.org/10.7554/eLife.15598.023

Taken together, these observations are consistent with a mechanism for coupling ATP hydrolysis in SecA to protein translocation that does not require a power stroke. In our proposed model (Figure 8 and Movie 1) the direction of random substrate diffusion (Brownian motion [Simon et al., 1992]) is biased by the action of the ATPase. In the absence of blockages, the polypeptide diffuses freely backwards and forwards through the closed (ADP-bound) channel (state (i) in Figure 8). When a region of polypeptide that cannot pass through reaches the channel entrance (state (ii) – block region as green circle), it triggers nucleotide exchange – probably *via* the 2HF (state (iii)). This causes a brief opening of the channel (iv), allowing the polypeptide to diffuse freely, before ATP is hydrolysed and the channel closes ((ii) or (v) depending on the position of the block). Back-diffusion is restricted because bulky regions on the periplasmic side do not trigger nucleotide exchange (vi): the whole scheme therefore acts as a ratchet promoting translocation. In essence, the bias for forward directionality arises because the energy transducing (ATP dependent) step – which resolves channel blockages – happens at the cytosolic, but not the periplasmic, surface. Moreover, the irreversibility of the initiation and completion steps would ensure forward directionality: once the signal sequence makes contact with the lipid bilayer (McKnight et al., 1991; Hizlan et al., 2012; Corey et al., 2016) it remains firmly fixed there, and is not cleaved off until most or all of the protein has crossed (Josefsson and Randall, 1981). This ensures that backward motion of the polypeptide does not lead to aborted transport.

It should be noted that we are limited by practical considerations in the size of peptide we can attach to the 2HF or the adjacent site on SecA. Nevertheless, its presence in the channel activates the NBS – the first demonstration of polypeptide-induced stimulation of the ATPase activity in solution (*i.e.* in the absence of a membrane). This selective response to differently sized peptides is very interesting: in the context of general protein transport, regions of folded secondary structure or stretches of polypeptide chain enriched in bulky amino acids are likely to occur (Park et al., 2014). These could be considerably larger than two tryptophans as in the peptide AWCWA. This, together with the fact that we see such variability in the width across LG with ATP bound (Figure 2C), suggests a degree of gradation in the mechanism: the larger and bulkier a region of substrate, the less likely it is to spontaneously cross the channel, but the more likely it is to induce nucleotide exchange. Indeed, at its core our model only requires a single open state to function: perhaps the most open state is reserved to conduct the largest substrates, after multiple rounds of ATP hydrolysis.

While previous models for translocation have postulated a substrate clamp within SecA, or large-scale conformational changes that physically push the substrate through the channel (van der Wolk et al., 1997; Erlandson et al., 2008; Bauer et al., 2014), the Brownian ratchet mechanism proposed here (Figure 8 and Video 1) requires only the conformational changes predicted by MD and verified experimentally by ensemble and single molecule FRET studies. Moreover, because much of the peptide is diffusing rather than being pushed, the proposed model retains scope for further acceleration by the PMF (Brundage et al., 1990), folding, or by the binding of periplasmic chaperones: these factors would also prevent backsliding, and thereby promote flux towards the periplasmic side of the membrane.

**Video 1.** Proposed Brownian ratchet mechanism for translocation The proposed Brownian ratchet model for protein translocation, as presented in Figure 8. Video produced by Nan Burston. **DOI:** http://dx.doi.org/10.7554/eLife.15598.024

The different open and closed states required for the model are represented in the various structures of the translocon determined in the presence or absence of translocation partners and engaged translocation substrate (Van den Berg et al., 2004; Zimmer et al., 2008; Park et al., 2014; Gogala et al., 2014; Pfeffer et al., 2015; Voorhees and Hegde, 2016). Indeed, a structure of SecYEG-SecA was very recently determined with a pseudo pre-protein within the channel (Li et al., 2016). With a narrow region of polypeptide (Ala-Gly-Gly) in the centre of the channel and ADP-BeF_x_ bound to the NBS, the LG closely resembles that of 3DIN (Zimmer et al., 2008) – or the part-open conformation reported here. The pore-ring is packed tightly around this sequence, suggesting (i) that with substrate bound, the complex might not reach the fully closed state described here (upon ATP hydrolysis), and (ii) that further opening (upon ATP binding) would be required to accommodate a larger region of substrate – as we propose here.

It is important to point out that the model does not rule out further conformational changes that are not observable on the timescale of our MD simulation, or resolved by the single molecule FRET analysis presented. It has been reported that the ATP-bound state of SecA binds more tightly to substrate than the ADP-bound state (Bauer et al., 2014). This could be evidence of an additional pre-protein clamp in SecA (Bauer and Rapoport, 2009), perhaps designed to prevent the (iv) → (ii) transition in Figure 8: such a possibility is not precluded by the data, and would represent a refinement of the model presented here. Alternately, it could form part of a proof-reading mechanism to prevent poorly translocated substrates from clogging and jamming the Sec machinery (Liang et al., 2012). This is known to be deleterious to cells, leading to degradation of SecY (van Stelten et al., 2009); SecA has indeed been reported to have unfolding activity (Arkowitz et al., 1993).

The proposed model (Figure 8 and Video 1) accounts for many previously noted properties of translocation: for example, the translocation intermediates sometimes observed at low ATP concentrations (van der Wolk et al., 1997) could be caused by regions of substrate with particularly high energy barriers to transit. It would also give rise to an approximately linear dependence of translocation rate on substrate length (Liang et al., 2009) – especially those with repeating sequences (Tomkiewicz et al., 2006) – while giving different rates for substrates with markedly different sequences (Sato et al., 1997). Furthermore, the fact that ATP-bound SecA is bound tightly to the channel, while the ADP-bound form can be released (Bauer et al., 2014), makes sense from the perspective of cellular homeostasis and membrane integrity: release of SecA from a wide open SecY channel would allow the leakage of ions and compromise the energy conserving capabilities of the plasma membrane.

The above mechanism represents a breakthrough in our understanding of protein export through the ubiquitous Sec complex. It also resembles a suggested mechanism for the eukaryotic Sec machinery (Matlack et al., 1999), but with ratcheting from the cytosolic rather than the distal side of the membrane, which compensates for the absence of ATP in the periplasm. Regulated constriction of the channel, therefore, may offer a unifying concept for protein transport more generally, *e.g.* in mitochondrial and chloroplast protein import. Furthermore, two-way communication between the ATPase NBS and the polymer binding site over distances ranging from 2 to 15 nm has been observed for several molecular motors such as RecA-like helicases (Mancini et al., 2004; Kainov et al., 2008) and AAA+ dynein (Kon et al., 2009). Thus, a common mechanism is emerging that may underlie many other systems responsible for the conversion of chemical energy into directional motion of proteins and nucleic acids.

## Materials and methods

### Molecular dynamics simulations

All simulations were run in GROMACS 4.6.4 (Berendsen et al., 1995). Models for the simulations were built using chains A, C, D and E of the crystal structure 3DIN (Zimmer et al., 2008) as starting coordinates, with missing loops added using Modeller (Sali and Blundell, 1993) and ADP-BeF_x_ replaced with either ADP or ATP (Piggot et al., 2012), or removed entirely. The protein was described using the OPLS-AA force field (Jorgensen et al., 1996) and embedded in a 512 united-atom POPC membrane (Ulmschneider and Ulmschneider, 2009), using the GROMACS utility g_membed (Wolf et al., 2010). Alternatively, the systems were described with Amber ff99SB-ILDN (Lindorff-Larsen et al., 2010) using a Lipid14 POPC membrane (Dickson et al., 2014). The protein-membrane structures were built into simulation boxes with periodic boundary conditions in all dimensions and solvated with explicit SPC water and sodium and chloride ions to a neutral charge and concentration of 0.15 M. In total there were approximately 230,000 atoms per simulation box. The systems were energy minimized using the steepest descents method over 2 x 5000 steps, then equilibrated for 1 ns using the NPT ensemble at 300 K with the Bussi-Donadio-Parrinello thermostat and semi-isotropic Parrinello-Rahman pressure coupling. Bond lengths were constrained using the LINCS method. Non-bonded interaction cut-offs were calculated using the Verlet method, with the neighbour search list updated every 20 steps. Long-range electrostatic interactions were calculated using the particle mesh Ewald method and a cut-off of 1.0 nm was applied for van der Waals and short range electrostatic interactions.

Molecular dynamics simulations were run with 2 fs integration time steps over 400–500 ns on the University of Bristol’s High Performance Computer, BlueCrystal, and where applicable extended to 1 µs on the UK HPC facility ARCHER. All simulations reached a steady state as judged by their root-mean-squared-deviation from the starting structure (Figure 2—figure supplement 1).

For the steered MD 'pulling' experiments, a penta-peptide was modelled into a representative closed or open post-200 ns-simulation structure, in the cavity between SecA and SecY (Figure 7—figure supplement 3B). The penta-peptide was either AGAGA, AFAFA or AWAWA, with the backbone coordinates identical between the peptides. Simulations were run with the peptide in place for 10 ns to allow the region to relax, before a constant force of 400 kJ mol−^1^ nm−^1^ was applied to the peptide in a z-axis direction. The simulations were run until the peptide passed through the channel (or for 100 ns). Ten repeats were run for each, and the transit times were plotted using a box and whisker plot, showing extremes, upper and lower quartiles and median.

### Protein preparation

Site-directed mutagenesis was performed using the QuikChange protocol (Agilent) and confirmed by sequencing.

SecYEG, SecA, SecA^A795C-FL^ (SecA*), SecY_x_EG-SecA_2HF_ and pOA were produced as described previously (Gold et al., 2007; Deville et al., 2011; Whitehouse et al., 2012). SecY**EG was produced in the same way as wild-type, then labelled for 45 mins on ice at 50 µM with 100 µM each of Alexa 488-C_5_-maleimide and Alexa 594-C_5_-maleimide (Invitrogen). The reactions were quenched with 10 mM DTT, and excess dye removed by gel filtration (Superdex-200, GE Healthcare, U.K.). Labelling efficiencies were between 75–90% for each dye, as determined using the manufacturer's quantification method and assuming a molar extinction coefficient of 70,820 cm^-1^ for SecYEG. Membranes expressing SecY_LG_EG, prepared as for wild-type, were oxidised with 1 mM copper phenanthroline prior to solubilisation in 1% n-Dodecyl β-D-Maltopyranoside (DDM). The samples were subjected to overnight batch-binding with Activated Thiol Sepharose 4B (GE Healthcare, U.K.), followed by centrifugation to remove uncross-linked protein. They were then treated by gel filtration as per the wild-type preparation.

SecA^G793C^, SecA^Q644C^ andSecA^R20C^ were prepared as for wild-type. Peptides (AGCGA, AFCFA or AWCWA) were purchased from Cambridge Research Biochemicals, all N-terminally acetylated and C-terminally amidated. Cross-linking was performed by incubating each single cysteine variant with a 20-fold excess of peptide and 10 mM oxidised glutathione for 1 hr on ice. Excess free peptide was removed by gel filtration (Superdex-200). The efficacy of this method was confirmed for the AWCWA peptide using reverse-phase HPLC (0–50% acetonitrile in 0.1% TFA) on an XBridge BEH300 C4 column (Waters, U.K.), following absorbance at 280 nm (Figure 7—figure supplement 2A).

PLs of *E. coli* polar lipid containing SecYEG were produced as described previously (Gold et al., 2007).

### ATPase and translocation assays

ATPase activities were determined as described previously (Gold et al., 2007). In brief, ATP consumption was coupled to NADH depletion using a pyruvate kinase/lactate dehydrogenase regenerating system in the presence of excess phosphoenol pyruvate, and the absorbance at 340 nm followed. Rates were determined by fitting to a straight line, and the slopes (in ∆A_340_.min−^1^) divided by the concentration of SecA and the molar extinction coefficient of NADH (6,220 M−^1^ at 340 nm) to give (M ATP).(M SecA)−^1^.min−^1^. All data fitting for ensemble experiments was performed using pro Fit (Quansoft).

Translocation efficiencies were determined using an in vitro translocation assay (Gold et al., 2007): after a 40 min translocation reaction at 25°C, all untranslocated material was degraded with protease K, and the translocated material quantified by western blotting.

### Equilibrium fluorescence measurements

Fluorescence spectra of SecY**EG PLs were measured on a Jobin Yvon Fluorolog (Horiba Scientific), exciting at 493 nm and measuring emission spectra. Measurements were performed in TKM buffer (20 mM Tris pH 7.5, 50 mM KCl, 2 mM MgCl_2_), starting with 50 nM SecY**EG, then adding sequentially 1 µM nucleotide-stripped SecA, 1 mM AMPPNP, ADP or ATP, and 0.7 µM pOA. At least 5 mins were left after each addition before measuring, and each spectrum was repeated three times to confirm that a steady state was reached. Data were corrected for dilution before plotting.

FRET measurements for SecY**EG in DDM were performed in TSG buffer (20 mM Tris pH 7.5, 130 mM NaCl, 10% glycerol) with 0.02% DDM and 2 mM MgCl_2_ on a Nanodrop 3300 Fluorospectrometer (Thermo Scientific, Waltham MA, ). 100 nM DDM-solubilised SecY**EG was measured alone, with 1 µM SecA, with SecA and 1 mM ADP, or with SecA and 1 mM AMPPNP. After excitation with blue light, fluorescence signals at 519 nm (donor; F_D_) and 617 nm (acceptor; F_A_) were measured, and FRET efficiencies calculated as F_A_ / (F_D_ + F_A_). For each data point, three repeats were taken and the average FRET efficiency used.

### Affinity assays

Affinity assays employing SecY**EG were performed by mixing 50 nM SecY**EG PLs with 500 µM ADP or AMPPNP in TKM buffer with varying amounts of SecA. Affinity assays reporting from SecA* were performed by mixing 10 nM SecA* in TSG buffer with 0.02% DDM, 2 mM MgCl_2_, 1 mM AMPPNP and varying concentrations of SecYEG. AMPPNP affinity assays were carried out by mixing varying concentrations of AMPPNP with pre-equilibrated 100 nM SecA and 500 nM SecYEG in the same buffer. DTT was either omitted or included at 100 mM; note that DTT has no effect on the interaction of SecA with wild-type SecYEG. Single channel fluorescence readings were taken at 522 nm and FRET readings at 519 nm and 617 nm, using a Nanodrop 3300 Fluorospectrometer with blue light excitation. The decreases in FRET or fluorescence were then plotted and fitted to a tight binding equation:

$$F=F_{0}-B_{max}\frac{E_{0}+s+K_{D}\;-\sqrt{{\left(E_{0}+s\;+K_{D}\right)}^{2}\;-\;4E_{0}s}}{2E_{0}}$$

where F is the fluorescence signal, F_0_ is signal in the absence of substrate, B_max_ is the amplitude of the fluorescence change, E_0_ is the concentration of the fixed binding partner, s is the substrate concentration and K_D_ is the dissociation constant.

### Single molecule FRET

SecY**EG was reconstituted into PLs with *E. coli* polar lipid to a final concentration of 1.5 nM, and extruded to 100 nm: at this concentration and size, most liposomes are expected to contain either 0 or 1 copy of SecY**EG (Deville et al., 2011). PLs were immobilised on a glass supported lipid bilayer and imaged with a TIRF microscope as described previously (Figure 5A). The buffer used was TKM with 1 mM 6-hydroxy-2,5,7,8-tetramethylchroman-2-carboxylic acid (TROLOX) and 71 mM β-mercaptoethanol as oxygen scavenging agents to extend florescent dye lifetimes. Immobilised samples were treated with 40 nM SecA and 1 mM ADP, 1 mM AMPPNP or 1 mM ATP and the ATP regeneration system used for ATPase assays, with or without 200 nM pOA. For the high SecA/high pOA sample (Figure 5G), 1 µM SecA and 700 nM pOA were used with 1 mM ATP and the ATP regeneration system. TIRF movies (200 ms frame rate) were taken from samples between 15 and 45 min after the addition of substrate.

The data processing flow is outlined in Figure 5—figure supplement 1A. The two channels of each image (Figure 5—figure supplement 1B) were aligned and fluorescence count traces (donor and acceptor) were extracted as described previously (Sharma et al., 2014) (Figure 5—figure supplement 1C). Since the system is essentially at equilibrium and the signals remain stationary we used the consecutive acceptor (first) and donor (second) single-step photobleaching events to identify complexes with exactly a single donor and single acceptor (Figure 5—figure supplement 1D). Only traces which exhibit an anti-correlated rise in donor signal upon acceptor photobleaching, a signature of single molecule FRET, are included in the analysis.

While this method discards FRET traces in which the donor photobleaches first, it eliminates the possibility of selecting traces with an ill-defined number of acceptor dyes in each immobilized PL. As the method provides access to the donor signal with acceptor present (before acceptor photobleaching) and absent (after acceptor photobleaching), it is a valid way to compute FRET efficiencies (Lakowicz, 2013) (Figure 5—figure supplement 1D). This method has the additional advantage that it does not need signal corrections for quantum yield changes or channel sensitivity. Note that while the acceptor signal is not directly used to calculate FRET efficiency its changes are employed to identify the smFRET traces and the relevant regions of the donor signal for data analysis (Figure 5—figure supplement 1C).

The experiments were repeated three times using independent PL preparations, then FRET values were collated as histograms (Figure 5—figure supplement 2A) and the three repeat histograms were averaged bin by bin to produce the final histogram with standard error for each bin (Figure 5B–G).

Singular value decomposition (SVD) of histograms for different conditions indicated than minimum of three components were necessary to describe the ATP data within experimental error (Figure 5—figure supplement 3B). Likewise, the 'ATP' individual repeat histograms can only be adequately decomposed into a minimum of three Gaussian components (Figure 5—figure supplements 2 and 3A). Using global fitting, the resulting amplitudes of the Gaussian peaks for the three independent repeats (Figure 5—figure supplement 1A) were subjected to ANOVA yielding mean amplitude and standard errors (SEM) (Figure 5—figure supplement 2B).

To measure dye anisotropy, 100 nM reconstituted SecY**EG was incubated for 5 min at 25°C alone or with 1 µM SecA and 1 mM ADP, AMPPNP or 1 mM ATP with the ATP regenerating system and 700 nM pOA. A Florolog (Horiba Jobin Yvon) was use to collect emission spectra for dye anisotropy. The samples were either excited at 492 nm and 510–650 nm emission was detected or excited at 590 nm and 610–700 nm emission recorded (increment 2 nm, integration time 1 s, slits 5 nm). Sensitivity correction (G factor) was determined using excitation polarisation parallel to emission propagation direction. Using the G correction, anisotropy was computed and averaged over the peak of each emission band (515–525 and 615–625 nm).

### Kinetic simulations

Simulations of interconversion between the closed (E_FRET _= 0.76) and open states (E_FRET _=0.45) were done using Gillespie discrete event algorithm (Gillespie, 1977; Gillespie, 1978) with time step 1 ms and dwell times set at 1/*k*_cat_ (*k*_cat_ = 0.27 s^-1^, the rate of ADP release) for the high FRET and 1/*k*_cleave_ (*k*_cleave_ = 11.5 s^-1^ the rate of ATP hydrolysis and phosphate release) for the low FRET state, respectively (Robson et al., 2009). Normally distributed noise was added to match the width of the high FRET peak (0.24) and the data was then averaged over 200 ms windows to emulate sampling during TIRF data collection. The algorithm and subsequent averaging was implemented in Matlab.

### Limited proteolysis

For trypsin digest analysis, 4 µg SecYEG samples were incubated with 0.75 µg.ml−^1^ (for proteins in DDM) or 6 µg.ml^-1^ (for proteins in PLs) sequencing grade porcine trypsin (Promega, Southampton, U.K.) in a total volume of 10 µl at room temperature for 20 min, then analysed by SDS-PAGE. In the case of PLs, the reactions were quenched with 200 mM NaOH prior to gel analysis. Bands were visualised and quantified either using an Odyssey Fc (LI-COR Biosciences, Lincoln, NE; for Coomassie stained gels) or a Typhoon FLA 9500 (GE Healthcare) and ImageJ (for fluorescence scans).

### Stopped flow fluorescence measurements of ADP off rates

0.6 µM SecA with or without 2.4 µM SecYEG in either PLs (for experiments using SecY_LG_EG) or 0.02% w/v DDM (wild-type SecYEG) were pre-mixed with 1 mM ADP to equilibrate and produce an ADP-bound state of SecA. This was mixed rapidly (<2 ms) with an equal volume of 25 µM MANT-ADP (Sigma Aldrich, St. Louis, MO) in the same buffer on a KinetAsyst SF-61SX2 stopped-flow (Hi-Tech). Tryptophan fluorescence was excited at 296 nm and emission was recorded using a 399 nm longpass filter; this allows monitoring of FRET between the tryptophans in SecA and the MANT moiety upon SecA binding. Because the off-rate for ADP << the on-rate for MANT-ADP, fitting to a single exponential yields the rate of ADP release (Robson et al., 2009).

Note that for experiments in PLs and with crosslinked SecY_x_EG-SecA_2HF_, a double exponential is required to fit the data. In the case of PLs (Figure 6D), this is likely a complication caused by the presence of lipids. For ease of comparison, we simply normalised the plots to give a total amplitude of 1 and rather than attempting to extract potentially spurious individual rates, we simply estimated the time taken to reach 50% completion (t_1/2_). For SecY_x_EG-SecA_2HF_ (Figure 7C), we reasoned that SecY_x_EG-SecA_2HF_ is inevitably contaminated with some free SecA^A795C ^(Whitehouse et al., 2012), so the populations most likely correspond to a mix of SecY_x_EG-SecA_2HF_ and SecA^A795C^. We confirmed this by treating the complex with 100 mM DTT, which yields a single exponential rate of ADP release (Figure 7C, turquoise line). To fit the *k*_off_ for SecY_x_EG-SecA_2HF_, we therefore fixed one exponential rate to that of the fully reduced complex, and allowed the other, along with both amplitudes, to float freely.

## Additional files

### Major datasets

The following previously published datasets were used:

van den Berg B, Clemons Jr WM, Collinson I, Modis Y, Hartmann E, Harrison SC, Rapoport TA,2004,The structure of a protein conducting channel,http://www.rcsb.org/pdb/explore/explore.do?structureId=1RHZ,Publicly available at the RCSB Protein Data Bank (accession no. 1RHZ)

Zimmer J, Nam Y, Rapoport TA,2008,Crystal structure of the protein-translocation complex formed by the SecY channel and the SecA ATPase,http://www.rcsb.org/pdb/explore/explore.do?structureId=3DIN,Publicly avaialble at the RCSB Protein Data Bank (accession no. 3DIN)

## References

[bib1] Akimaru J, Matsuyama S, Tokuda H, Mizushima S (1991). Reconstitution of a protein translocation system containing purified SecY, SecE, and SecA from Escherichia coli. Proceedings of the National Academy of Sciences of the United States of America.

[bib2] Arkowitz RA, Joly JC, Wickner W (1993). Translocation can drive the unfolding of a preprotein domain. The EMBO Journal.

[bib3] Banroques J, Doère M, Dreyfus M, Linder P, Tanner NK (2010). Motif III in superfamily 2 "helicases" helps convert the binding energy of ATP into a high-affinity RNA binding site in the yeast *dead-box protein ded1*. Journal of Molecular Biology.

[bib4] Bauer BW, Rapoport TA (2009). Mapping polypeptide interactions of the SecA ATPase during translocation. Proceedings of the National Academy of Sciences of the United States of America.

[bib5] Bauer BW, Shemesh T, Chen Y, Rapoport TA (2014). A "push and slide" mechanism allows sequence-insensitive translocation of secretory proteins by the SecA ATPase. Cell.

[bib6] Berendsen HJC, van der Spoel D, van Drunen R (1995). GROMACS: A message-passing parallel molecular dynamics implementation. Computer Physics Communications.

[bib7] Bessonneau P, Besson V, Collinson I, Duong F (2002). The SecYEG preprotein translocation channel is a conformationally dynamic and dimeric structure. The EMBO Journal.

[bib8] Breyton C, Haase W, Rapoport TA, Kühlbrandt W, Collinson I (2002). Three-dimensional structure of the bacterial protein-translocation complex SecYEG. Nature.

[bib9] Brundage L, Hendrick JP, Schiebel E, Driessen AJ, Wickner W (1990). The purified E. coli integral membrane protein SecY/E is sufficient for reconstitution of SecA-dependent precursor protein translocation. Cell.

[bib10] Cannon KS, Or E, Clemons WM, Shibata Y, Rapoport TA (2005). Disulfide bridge formation between SecY and a translocating polypeptide localizes the translocation pore to the center of SecY. The Journal of Cell Biology.

[bib11] Collinson I, Corey RA, Allen WJ (2015). Channel crossing: how are proteins shipped across the bacterial plasma membrane?. Philosophical Transactions of the Royal Society of London. Series B, Biological Sciences.

[bib12] Corey RA, Allen WJ, Komar J, Masiulis S, Menzies S, Robson A, Collinson I (2016). Unlocking the bacterial SecY translocon. Structure.

[bib13] Deville K, Gold VA, Robson A, Whitehouse S, Sessions RB, Baldwin SA, Radford SE, Collinson I (2011). The oligomeric state and arrangement of the active bacterial translocon. The Journal of Biological Chemistry.

[bib14] Dickson CJ, Madej BD, Skjevik AA, Betz RM, Teigen K, Gould IR, Walker RC (2014). Lipid14: The amber lipid force field. Journal of Chemical Theory and Computation.

[bib15] du Plessis DJ, Berrelkamp G, Nouwen N, Driessen AJ (2009). The lateral gate of secyeg opens during protein translocation. The Journal of Biological Chemistry.

[bib16] Economou A, Wickner W (1994). SecA promotes preprotein translocation by undergoing ATP-driven cycles of membrane insertion and deinsertion. Cell.

[bib17] Eisenberg D, McLachlan AD (1986). Solvation energy in protein folding and binding. Nature.

[bib18] Erlandson KJ, Miller SB, Nam Y, Osborne AR, Zimmer J, Rapoport TA (2008). A role for the two-helix finger of the SecA ATPase in protein translocation. Nature.

[bib19] Fisher AJ, Smith CA, Thoden JB, Smith R, Sutoh K, Holden HM, Rayment I (1995). X-ray structures of the myosin motor domain of dictyostelium discoideum complexed with MgADP.BeFx and MgADP.AIF4-. Biochemistry.

[bib20] Gillespie DT (1977). Exact stochastic simulation of coupled chemical reactions. The Journal of Physical Chemistry.

[bib21] Gillespie DT (1978). Monte Carlo simulation of random walks with residence time dependent transition probability rates. Journal of Computational Physics.

[bib22] Gogala M, Becker T, Beatrix B, Armache JP, Barrio-Garcia C, Berninghausen O, Beckmann R (2014). Structures of the Sec61 complex engaged in nascent peptide translocation or membrane insertion. Nature.

[bib23] Gold VA, Robson A, Clarke AR, Collinson I (2007). Allosteric regulation of SecA: Magnesium-mediated control of conformation and activity. The Journal of Biological Chemistry.

[bib24] Gold VA, Whitehouse S, Robson A, Collinson I (2013). The dynamic action of SecA during the initiation of protein translocation. The Biochemical Journal.

[bib25] Gouridis G, Karamanou S, Gelis I, Kalodimos CG, Economou A (2009). Signal peptides are allosteric activators of the protein translocase. Nature.

[bib26] Görlich D, Hartmann E, Prehn S, Rapoport TA (1992). A protein of the endoplasmic reticulum involved early in polypeptide translocation. Nature.

[bib27] Hall MC, Ozsoy AZ, Matson SW (1998). Site-directed mutations in motif VI of escherichia coli DNA helicase II result in multiple biochemical defects: Evidence for the involvement of motif VI in the coupling of atpase and DNA binding activities via conformational changes. Journal of Molecular Biology.

[bib28] Hardesty B, Kramer G (2001). Folding of a nascent peptide on the ribosome. Progress in Nucleic Acid Research and Molecular Biology.

[bib29] Hartl FU, Lecker S, Schiebel E, Hendrick JP, Wickner W (1990). The binding cascade of SecB to SecA to SecY/E mediates preprotein targeting to the E. coli Plasma membrane. Cell.

[bib30] Henry GD, Maruta S, Ikebe M, Sykes BD (1993). Observation of multiple myosin subfragment 1-adp-fluoroberyllate complexes by 19F NMR spectroscopy. Biochemistry.

[bib31] Hizlan D, Robson A, Whitehouse S, Gold VA, Vonck J, Mills D, Kühlbrandt W, Collinson I (2012). Structure of the secy complex unlocked by a preprotein mimic. Cell Reports.

[bib32] Hunt JF, Weinkauf S, Henry L, Fak JJ, McNicholas P, Oliver DB, Deisenhofer J (2002). Nucleotide control of interdomain interactions in the conformational reaction cycle of SecA. Science.

[bib33] Ito K, Wittekind M, Nomura M, Shiba K, Yura T, Miura A, Nashimoto H (1983). A temperature-sensitive mutant of E. coli exhibiting slow processing of exported proteins. Cell.

[bib34] Jorgensen WL, Maxwell DS, Tirado-Rives J (1996). Development and testing of the OPLS all-atom force field on conformational energetics and properties of organic liquids. Journal of the American Chemical Society.

[bib35] Josefsson LG, Randall LL (1981). Different exported proteins in E. coli show differences in the temporal mode of processing in vivo. Cell.

[bib36] Kainov DE, Mancini EJ, Telenius J, Lísal J, Grimes JM, Bamford DH, Stuart DI, Tuma R (2008). Structural basis of mechanochemical coupling in a hexameric molecular motor. The Journal of Biological Chemistry.

[bib37] Karamanou S, Vrontou E, Sianidis G, Baud C, Roos T, Kuhn A, Politou AS, Economou A (1999). A molecular switch in SecA protein couples ATP hydrolysis to protein translocation. Molecular Microbiology.

[bib38] Kon T, Imamula K, Roberts AJ, Ohkura R, Knight PJ, Gibbons IR, Burgess SA, Sutoh K (2009). Helix sliding in the stalk coiled coil of dynein couples ATPase and microtubule binding. Nature Structural & Molecular Biology.

[bib39] Lakowicz JR (2013). Principles of Fluorescence Spectroscopy.

[bib40] Li L, Park E, Ling J, Ingram J, Ploegh H, Rapoport TA (2016). Crystal structure of a substrate-engaged SecY protein-translocation channel. Nature.

[bib41] Liang FC, Bageshwar UK, Musser SM (2009). Bacterial sec protein transport is rate-limited by precursor length: A single turnover study. Molecular Biology of the Cell.

[bib42] Liang FC, Bageshwar UK, Musser SM (2012). Position-dependent effects of polylysine on sec protein transport. The Journal of Biological Chemistry.

[bib43] Lill R, Cunningham K, Brundage LA, Ito K, Oliver D, Wickner W (1989). SecA protein hydrolyzes ATP and is an essential component of the protein translocation ATPase of Escherichia coli. The EMBO Journal.

[bib44] Lindorff-Larsen K, Piana S, Palmo K, Maragakis P, Klepeis JL, Dror RO, Shaw DE (2010). Improved side-chain torsion potentials for the amber ff99sb protein force field. Proteins.

[bib45] Lu J, Deutsch C (2005). Secondary structure formation of a transmembrane segment in kv channels. Biochemistry.

[bib46] Mancini EJ, Kainov DE, Grimes JM, Tuma R, Bamford DH, Stuart DI (2004). Atomic snapshots of an RNA packaging motor reveal conformational changes linking ATP hydrolysis to RNA translocation. Cell.

[bib47] Mao C, Cheadle CE, Hardy SJ, Lilly AA, Suo Y, Sanganna Gari RR, King GM, Randall LL (2013). Stoichiometry of SecYEG in the active translocase of Escherichia coli varies with precursor species. Proceedings of the National Academy of Sciences of the United States of America.

[bib48] Matlack KE, Misselwitz B, Plath K, Rapoport TA (1999). Bip acts as a molecular ratchet during posttranslational transport of prepro-alpha factor across the ER membrane. Cell.

[bib49] McKnight CJ, Stradley SJ, Jones JD, Gierasch LM (1991). Conformational and membrane-binding properties of a signal sequence are largely unaltered by its adjacent mature region. Proceedings of the National Academy of Sciences of the United States of America.

[bib50] Nouwen N, Berrelkamp G, Driessen AJ (2009). Charged amino acids in a preprotein inhibit SecA-dependent protein translocation. Journal of Molecular Biology.

[bib51] Osborne AR, Rapoport TA (2007). Protein translocation is mediated by oligomers of the secy complex with one SecY copy forming the channel. Cell.

[bib52] Park E, Ménétret JF, Gumbart JC, Ludtke SJ, Li W, Whynot A, Rapoport TA, Akey CW (2014). Structure of the SecY channel during initiation of protein translocation. Nature.

[bib53] Pause A, Méthot N, Sonenberg N (1993). The HRIGRXXR region of the DEAD box RNA helicase eukaryotic translation initiation factor 4A is required for RNA binding and ATP hydrolysis. Molecular and Cellular Biology.

[bib54] Peyser YM, Ajtai K, Burghardt TP, Muhlrad A (2001). Effect of ionic strength on the conformation of myosin subfragment 1-nucleotide complexes. Biophysical Journal.

[bib55] Pfeffer S, Burbaum L, Unverdorben P, Pech M, Chen Y, Zimmermann R, Beckmann R, Förster F (2015). Structure of the native sec61 protein-conducting channel. Nature Communications.

[bib56] Phan BC, Peyser YM, Reisler E, Muhlrad A (1997). Effect of complexes of ADP and phosphate analogs on the conformation of the Cys707-Cys697 region of myosin subfragment 1. European Journal of Biochemistry / FEBS.

[bib57] Piggot TJ, Sessions RB, Burston SG (2012). Toward a detailed description of the pathways of allosteric communication in the groel chaperonin through atomistic simulation. Biochemistry.

[bib58] Robson A, Gold VA, Hodson S, Clarke AR, Collinson I (2009). Energy transduction in protein transport and the ATP hydrolytic cycle of SecA. Proceedings of the National Academy of Sciences of the United States of America.

[bib59] Sali A, Blundell TL (1993). Comparative protein modelling by satisfaction of spatial restraints. Journal of Molecular Biology.

[bib60] Sato K, Mori H, Yoshida M, Tagaya M, Mizushima S (1997). Short hydrophobic segments in the mature domain of proompa determine its stepwise movement during translocation across the cytoplasmic membrane of escherichia coli. The Journal of Biological Chemistry.

[bib61] Schulze RJ, Komar J, Botte M, Allen WJ, Whitehouse S, Gold VA, Lycklama A Nijeholt JA, Huard K, Berger I, Schaffitzel C, Collinson I (2014). Membrane protein insertion and proton-motive-force-dependent secretion through the bacterial holo-translocon SecYEG-SecDF-yajc-yidc. Proceedings of the National Academy of Sciences of the United States of America.

[bib62] Sharma A, Leach RN, Gell C, Zhang N, Burrows PC, Shepherd DA, Wigneshweraraj S, Smith DA, Zhang X, Buck M, Stockley PG, Tuma R (2014). Domain movements of the enhancer-dependent sigma factor drive DNA delivery into the RNA polymerase active site: Insights from single molecule studies. Nucleic Acids Research.

[bib63] Simon SM, Peskin CS, Oster GF (1992). What drives the translocation of proteins?. Proceedings of the National Academy of Sciences of the United States of America.

[bib64] Tomkiewicz D, Nouwen N, van Leeuwen R, Tans S, Driessen AJ (2006). SecA supports a constant rate of preprotein translocation. The Journal of Biological Chemistry.

[bib65] Ulmschneider JP, Ulmschneider MB (2009). United Atom Lipid Parameters for Combination with the Optimized Potentials for Liquid Simulations All-Atom Force Field. Journal of Chemical Theory and Computation.

[bib66] Van den Berg B, Clemons WM, Collinson I, Modis Y, Hartmann E, Harrison SC, Rapoport TA (2004). X-ray structure of a protein-conducting channel. Nature.

[bib67] van der Wolk JP, de Wit JG, Driessen AJ (1997). The catalytic cycle of the escherichia coli SecA ATPase comprises two distinct preprotein translocation events. The EMBO Journal.

[bib68] van Stelten J, Silva F, Belin D, Silhavy TJ (2009). Effects of Antibiotics and a Proto-Oncogene Homolog on Destruction of Protein Translocator SecY. Science.

[bib69] Voorhees RM, Hegde RS (2016). Structure of the Sec61 channel opened by a signal sequence. Science.

[bib70] Whitehouse S, Gold VA, Robson A, Allen WJ, Sessions RB, Collinson I (2012). Mobility of the SecA 2-helix-finger is not essential for polypeptide translocation via the SecYEG complex. The Journal of Cell Biology.

[bib71] Wolf MG, Hoefling M, Aponte-Santamaría C, Grubmüller H, Groenhof G (2010). g_membed: Efficient insertion of a membrane protein into an equilibrated lipid bilayer with minimal perturbation. Journal of Computational Chemistry.

[bib72] Zimmer J, Nam Y, Rapoport TA (2008). Structure of a complex of the ATPase SecA and the protein-translocation channel. Nature.

